# Integrative multi-omics reveals the POSTN^+^ CAF–APOE^+^ macrophage axis drives immunosuppression and progression in prostate cancer

**DOI:** 10.3389/fimmu.2026.1845274

**Published:** 2026-08-10

**Authors:** Yangzhou Liu, Aochu Liu, Xinglai Dai, Jianpeng Zhang, Run Shi, Yingjian Wang, Guohua Zeng

**Affiliations:** 1 Department of Urology, The First Affiliated Hospital of Guangzhou Medical University, Guangzhou, China; 2 Guangdong Provincial Key Laboratory of Urological Diseases, Guangzhou, China; 3 Guangdong Engineering Research Center of Urinary Minimally Invasive Surgery Robot and Intelligent Equipment, Guangzhou, China; 4 Guangzhou Institute of Urology, Guangzhou Medical University, Guangzhou, China; 5 School of Basic Medical Sciences, Guangzhou Medical University, Guangzhou, Guangdong, China; 6 Department of Urology, The First Affiliated Hospital of Zhengzhou University, Zhengzhou, Henan, China; 7 Center of Digital Health, Berlin Institute of Health at Charité - Universitätsmedizin Berlin, Berlin, Germany; 8 Department of Oncology, The First Affiliated Hospital of Nanjing Medical University, Nanjing, China

**Keywords:** cancer-associated fibroblasts, immunotherapy resistance, periostin, prostate cancer, single-cell RNA sequencing, stromal-immune crosstalk, tumor microenvironment, tumor-associated macrophages

## Abstract

**Background:**

The progression of prostate cancer to lethal castration-resistant (CRPC) and metastatic (mCRPC) stages is driven by a profoundly remodeled tumor microenvironment (TME). However, the identity of key stromal cell populations, their developmental dynamics, and their precise crosstalk with immune cells remain incompletely understood, limiting our ability to target the TME therapeutically.

**Methods:**

We integrated single-cell RNA sequencing (scRNA-seq) data from 222,529 cells across 10 studies, encompassing normal prostate, primary tumors, CRPC, and mCRPC. Fibroblast heterogeneity was resolved using unsupervised clustering, trajectory inference (Monocle2), and regulon analysis (pySCENIC). Intercellular communication was deciphered using CellChat. Spatial transcriptomic data were integrated via CellTrek and SpaGene for validation. Clinical associations were evaluated in multiple bulk transcriptomic cohorts (e.g., TCGA-PRAD, IMvigor210) and extended to a pan-cancer atlas of 11 tumor types.

**Results:**

We identified a distinct POSTN^+^ CAF subset that was progressively enriched in advanced disease. Trajectory analysis positioned POSTN^+^ CAFs as a progenitor-like state potentially transitioning toward inflammatory or contractile subtypes, with STAT1-centered regulon activity associated with this process. POSTN^+^ CAFs created an extracellular matrix (ECM)-remodeled, immune-excluded niche, correlated with elevated T-cell exclusion scores. We further uncovered a predicted communication axis where POSTN^+^ CAFs are computationally inferred to interact with M2-like, immunoregulatory APOE^+^ macrophages via the MDK-NCL ligand-receptor pair. The co-occurrence of POSTN^+^ cancer-associated fibroblasts and APOE^+^ macrophages correlated with worse patient outcomes. In the IMvigor210 urothelial carcinoma cohort (as indirect cross-cancer evidence), high co-infiltration was associated with diminished anti-PD-L1 response, although this finding requires validation in prostate cancer-specific cohorts. Exploratory pan-cancer analysis suggested conserved enrichment and adverse prognostic impact of this stromal-immune axis across diverse tumor types, though tissue-specific context should be considered.

**Conclusions:**

Our integrated single-cell atlas defines a critical POSTN^+^ CAF–APOE^+^ macrophage unit that is associated with a fibrotic and immunosuppressive TME in advanced prostate cancer. If functionally validated, targeting this stromal-immune crosstalk axis could represent a promising therapeutic strategy to remodel the TME and overcome treatment resistance, although this remains speculative at present.

## 1 Introduction

Prostate cancer remains a leading cause of cancer-related mortality in men worldwide (1–3). Its clinical progression, from localized disease to lethal castration-resistant (CRPC) and metastatic (mCRPC) stages, is driven not only by malignant epithelial cells but also by profound remodeling of the tumor microenvironment (TME) (4). The TME is a complex ecosystem comprising stromal cells, immune infiltrates, and extracellular matrix (ECM), which collectively influence tumor growth, therapeutic response, and disease outcome (5–7). Among stromal components, cancer-associated fibroblasts (CAFs) are recognized as key architects of the TME, modulating ECM structure, immune cell recruitment and function, and metastatic potential (8–10). However, the heterogeneity of CAFs, their developmental trajectories, and their precise functional crosstalk with immune cells in prostate cancer progression are not fully resolved.

Similarly, the immune landscape of prostate cancer is characterized by general immunosuppression and poor response to immune checkpoint inhibitors (3, 11, 12). Tumor-associated macrophages (TAMs), particularly those with an M2-like, immunosuppressive phenotype, are abundant in prostate tumors and correlate with adverse prognosis (13, 14). Emerging evidence suggests dynamic interplay between specific CAF subsets and immune populations in shaping an immune-excluded or suppressive milieu (15, 16). Yet, a systematic, high-resolution dissection of the stromal-immune architecture and the molecular circuits governing their interaction across the spectrum of prostate cancer disease states is lacking.

Recent advances in single-cell RNA sequencing (scRNA-seq) offer an unprecedented opportunity to deconvolve cellular heterogeneity and intercellular communication within the TME at scale (17). While prior studies have profiled specific stages of prostate cancer, an integrated analysis encompassing normal tissue, primary tumors, and advanced disease is necessary to map the continuum of TME evolution. Specifically, identifying dominant CAF subpopulations that emerge during progression, defining their transcriptional programs and spatial organization, and elucidating their privileged communication pathways with immune cells are critical steps toward understanding therapy resistance and identifying novel therapeutic targets (18).

In this study, we constructed a comprehensive single-cell transcriptomic atlas of prostate cancer by integrating data from 222,529 cells across normal tissue, primary tumors, CRPC, and mCRPC. This integrated framework enabled us to systematically characterize the cellular composition, transcriptional states, and dynamic remodeling of the TME throughout disease progression. We focused on resolving CAF heterogeneity and identified a distinct subset marked by high expression of Periostin (POSTN) that is progressively enriched in advanced disease. We further delineated a transcriptional trajectory suggesting POSTN^+^ CAFs as a potential progenitor-like state, uncovered a STAT1-centered regulatory program, and characterized their role in constructing an ECM-remodeled, immune-excluded niche. By investigating stromal-immune crosstalk, we revealed a preferential interaction between POSTN+ CAFs and M2-polarized APOE+ macrophages mediated by the MDK-NCL signaling axis. The co-enrichment of these two populations was associated with poor clinical outcome and reduced benefit from immunotherapy in prostate cancer. Finally, we extended our analysis across multiple cancer types, demonstrating the pan-cancer relevance of the POSTN+ CAF–APOE+ macrophage axis in TME organization and patient prognosis. Our work provides a high-resolution landscape of prostate cancer TME, unveils a critical stromal-immune unit driving disease progression, and identifies potential targets for disrupting this tumor-promoting microenvironment.

## 2 Materials and methods

### 2.1 Data collection

A prostate cancer single-cell RNA-seq cohort was constructed by integrating 10 publicly available human PCa scRNA-seq datasets from Zhao et al. (19). We selected this integrated resource as our discovery cohort for the following reasons: (i) it represents the largest curated, harmonized prostate cancer scRNA-seq dataset available at the time of analysis, encompassing 93 samples across the full disease spectrum from normal prostate to mCRPC; (ii) the original study performed rigorous quality control and batch correction, providing a reliable foundation for downstream analysis; (iii) the dataset includes matched clinical annotations enabling disease-stage stratification; and (iv) the public availability of processed data ensured reproducibility. This integrated cohort comprises 93 samples spanning distinct disease stages and serves as the discovery cohort for subsequent analyses. Bulk transcriptomic data for the following nine prostate cancer cohorts—Cambridge, CancerMap, CHPCMA, CPCGene, DKFZ, GSE70769, MSKCC2010, Taylor, and TCGA-PRAD—were downloaded from the Prostate Cancer Database (PCaDB) at http://bioinfo.jialab-ucr.org/PCaDB/ (20). Spatial transcriptomics data (GSE230282 and GSE278936) were obtained from the GEO database.

### 2.2 Single-cell RNA sequencing analysis

Downstream processing of single-cell RNA-seq data was carried out using the Seurat R package (v4.3.0). Cells exhibiting fewer than 500 UMIs or mitochondrial gene content exceeding 15% were excluded, and probable doublets were detected via DoubletFinder (v2.0.4). Inter-sample batch effects were harmonized with the Harmony algorithm. Batch correction quality was assessed by visual inspection of UMAP embeddings before and after Harmony integration, evaluation of mixing metrics (kBET batch-mixing score), and confirmation that biological signals (e.g., disease-stage separation, cell-type clustering) were preserved while technical dataset-specific clustering was eliminated. A set of 2000 highly variable genes was identified using FindVariableFeatures for subsequent principal component analysis (PCA). Cell clustering was conducted based on the top 50 principal components, employing FindNeighbors and FindClusters, and visualized through UMAP. Canonical markers facilitated the annotation of principal cell types, including Epithelial cells (EPCAM, KRT15, AR, KRT17), T cells (CD3D, CD3E, NKG7, CD3G), Myeloid cells (C1QA, C1QB, CD14, CD68), Fibroblasts (COL1A1, DCN, LUM, ACTA2), Endothelial cells (VWF, CLDN5, ENG, PECAM1), Mast cells (CPA3, TPSAB1, TPSB2, KIT), and B cells (MS4A1, CD19, CD79A, CD79B). Differentially expressed genes (DEGs) were determined with FindAllMarkers, applying thresholds of min.pct = 0.25, logfc.threshold = 0.25, and only.pos = TRUE. Statistical significance was assessed using the Wilcoxon rank-sum test with Benjamini–Hochberg adjustment. These quality control metrics, together with the preservation of known biological cell-type separation (e.g., distinct clustering of epithelial cells, fibroblasts, and immune cells), support the robustness of our integrated dataset for downstream comparative analyses.

### 2.3 Assessment of gene signature activity

To evaluate pathway and signature activity at the single-cell level, the AUCell package (v1.20.2) was utilized. Genes were ranked per cell with the AUCell_buildRankings function, and enrichment scores were computed. Differences in signature activity between groups were compared using a two-tailed Wilcoxon rank-sum test, followed by false discovery rate (FDR) correction. All applied gene sets are provided in **Supplementary Table 1**.

### 2.4 Pathway and cytokine signaling analysis

CAF-related pathway activities were inferred using PROGENy. Scores were computed with run_aucell from the progeny (v1.24.0) package, based on the PROGENy reference network. The top 1000 target genes were used following standard single-cell recommendations.

### 2.5 Functional enrichment analysis

Biological pathway analyses, including pathway enrichment and gene set enrichment analysis (GSEA), were performed using the clusterProfiler package (v4.6.2) with KEGG pathway databases. Terms displaying an adjusted p-value below 0.05 were regarded as statistically significant.

### 2.6 Inference of cellular differentiation trajectories

The differentiation path of cancer-associated fibroblasts (CAFs) was modeled using Monocle2 (v2.26.0). The top 100 differentially expressed genes (DEGs) across CAF subsets were selected for trajectory construction. Data dimensionality was reduced via the reduceDimension function (method = DDRTree, max_components = 2), and genes exhibiting significant correlation with inferred pseudotime were determined using differentialGeneTest. Branch-dependent expression programs were analyzed with the BEAM module, and resulting dynamics were visualized using plot_genes_branched_pseudotime.

### 2.7 Inference of transcription factor activity

Transcriptional regulon inference was conducted with pySCENIC, which assesses regulon activity by integrating co-expression patterns and cis-regulatory motif analysis. Transcription factors associated with specific cell subtypes were identified via the Wilcoxon test, and regulon specificity was quantified using Jensen–Shannon divergence with the philentropy package (v0.6.0).

### 2.8 Analysis of intercellular communication

To investigate potential ligand–receptor interactions between cell populations, we applied the CellChat tool (v2.2.0) to single-cell RNA-seq data, utilizing CellChatDB.human as the reference interaction database. A significance threshold (P < 0.05) was used to infer biologically relevant cell–cell communication events.

### 2.9 Determination of cellular CNV levels

To assess chromosomal copy number alterations at the single-cell level, we utilized the InferCNV algorithm (v1.16.0). This method performs copy number karyotyping to differentiate non-malignant cells from those exhibiting aneuploidy. Normal epithelial cells served as the reference baseline to determine whether other cell populations displayed significant, large-scale copy number variations. This approach was also applied to further delineate malignant cells within the broader EPC (epithelial cell) population.

### 2.10 CytoTRACE analysis

We applied CytoTRACE (v0.3.3) to scRNA-seq data to assess the differentiation status of cell subsets. This algorithm ranks cells based on their developmental potential, allowing comparative analysis of differentiation states across populations.

### 2.11 Deconvolution of bulk transcriptomic data

Cellular composition within bulk RNA-sequencing and microarray profiles was inferred through BayesPrism. Custom signature matrices were generated from the single-cell RNA-seq reference data. For RNA-seq data, quartile normalization was disabled, while it was applied to microarray datasets. Resulting cell-type proportions were evaluated for correlation using Spearman’s rank test and visualized via the corrplot package (v1.6.10).

### 2.12 Clinical outcome and treatment response analysis

Patient overall survival was assessed using the survival package in R. Hazard ratios (HRs) and 95% confidence intervals (CIs) were derived from Cox proportional hazards regression models. Survival differences between groups were visualized using Kaplan–Meier curves generated with survfit and statistically compared via the log-rank test. Differences in response rates to immune checkpoint blockade (ICB) therapy were evaluated with the chi-square test.

### 2.13 Spatial transcriptomics analysis

Spatial gene-spot matrices derived from ST and Visium data were processed using Seurat (v4.3.0). Spots expressing fewer than 200 genes or genes detected in fewer than three spots were filtered out. Data were normalized with LogVMR, and dimensionality reduction was performed using PCA (first 30 PCs). Spatial enhancement was conducted with the BayesSpace package (v1.19.4) using the spatialEnhance and enhanceFeatures functions.

To spatially localize cell clusters identified from integrated single-cell RNA sequencing, CellTrek (v0.0.94) was used to jointly model scRNA-seq and ST expression matrices, incorporating spatial coordinates for precise mapping. Cell-type-specific transcriptional signatures, derived from single-cell data, were subsequently projected onto the spatially resolved dataset using the AddModuleScore function in Seurat and visualized through SpatialFeaturePlot. Lastly, spatial gene-expression patterns were systematically categorized using the SpaGene algorithm, which identified ten distinct expression modules per specimen. Module similarity was quantified with the Jaccard index and graphically represented via the ComplexHeatmap package (v2.14.0). It should be noted that CellTrek-based mapping provides probabilistic cell-type assignments at each spatial spot rather than definitive single-cell localization. Spatial gene-program correlation (e.g., via SpaGene) infers co-expression patterns within tissue neighborhoods but does not establish direct ligand-receptor binding or intercellular contact.

### 2.14 Tumor boundary identification

To delineate tumor boundaries, we employed SpaCET, an R package designed for spatial transcriptomic analysis in cancer. The package leverages a curated dictionary of gene expression signatures from common malignancies to infer cancer cell abundance and utilizes a constrained linear regression model to correct for local tissue density. Based on a comprehensive atlas of non-malignant cell types, SpaCET further quantifies stromal and immune lineage scores, thereby enabling precise spatial mapping of tumor margins and the adjacent microenvironment.

### 2.15 Tissue sample processing and western blotting

A total of 12 pairs of prostate cancer and adjacent normal tissue specimens were collected to validate the findings. This study was approved by the Ethics Committee of the First Affiliated Hospital of Guangzhou Medical University (approve number: ES-2024-K058-01), and written informed consent was obtained from all patients.

Fresh prostate cancer specimens and matched normal adjacent tissues were physically disrupted. Total protein was isolated using RIPA buffer containing both protease and phosphatase inhibitors (Beyotime Biotechnology). Protein concentration was measured with a BCA assay kit (Beyotime Biotechnology). Equal amounts of protein were separated by electrophoresis on 10% SDS-PAGE gels and then transferred onto PVDF membranes (Bio-Rad Laboratories). After blocking with 5% skim milk for two hours at room temperature, membranes were probed with primary antibodies overnight at 4°C. Following three washes with TBST, membranes were incubated for one hour with DyLight 800-conjugated secondary antibodies (Abbkine) matching the primary antibody host species. Protein bands were visualized using an enhanced chemiluminescence detection system. Primary antibodies included rabbit anti-POSTN monoclonal antibody (1:1000, ab215199, Abcam) and rabbit anti-Tubulin polyclonal antibody (1:1000, 10094-1-AP, Proteintech) for loading control. Band intensity was quantified with ImageJ software (National Institutes of Health). POSTN expression was measured in whole tissue lysates, and therefore represents total tissue POSTN protein rather than CAF-specific expression.

### 2.16 Paraffin immunofluorescence

FFPE prostate cancer sections were deparaffinized, rehydrated, and treated with methanol containing 3% H_2_O_2_ to block endogenous peroxidase. Three sequential rounds of staining were performed, each comprising antigen retrieval (citrate buffer, pH 6.0, microwave, medium power, 15 min), blocking with 5% BSA, primary antibody incubation, HRP-conjugated secondary antibody incubation, and TSA. Between rounds, bound antibodies were stripped using the same citrate buffer/microwave conditions. Primary antibodies were applied in the order anti-POSTN (Proteintech, 66491-1-Ig, mouse monoclonal; CAFs), anti-CD68 (Proteintech, 25747-1-AP, rabbit polyclonal; macrophages), and anti-CD8a (Proteintech, 66868-1-Ig, mouse monoclonal; CD8^+^ T cells), each at a dilution optimized by titration. Signals were visualized using the PANO IHC kit (10004100100, Panovue, Beijing, China) with spectrally distinct PPD-tyramide reagents, and nuclei were counterstained with DAPI.

### 2.17 Statistical evaluation

All statistical evaluations were conducted using R software (v4.3.1). Correlation analyses were performed with Spearman’s rank test. Comparisons between groups were carried out using the Kruskal–Wallis test and Wilcoxon rank-sum test, with multiple testing adjustments applied via the Benjamini–Hochberg method. Statistical significance was defined as p < 0.05. All figures indicate the statistical unit (cells, patients, samples, spots, or cohorts) in the corresponding legends to ensure transparency of the analytical unit for each analysis.

## 3 Results

### 3.1 Single-cell landscape of prostate cancer reveals stromal and immune architecture of the tumor microenvironment

**Figure 1:**
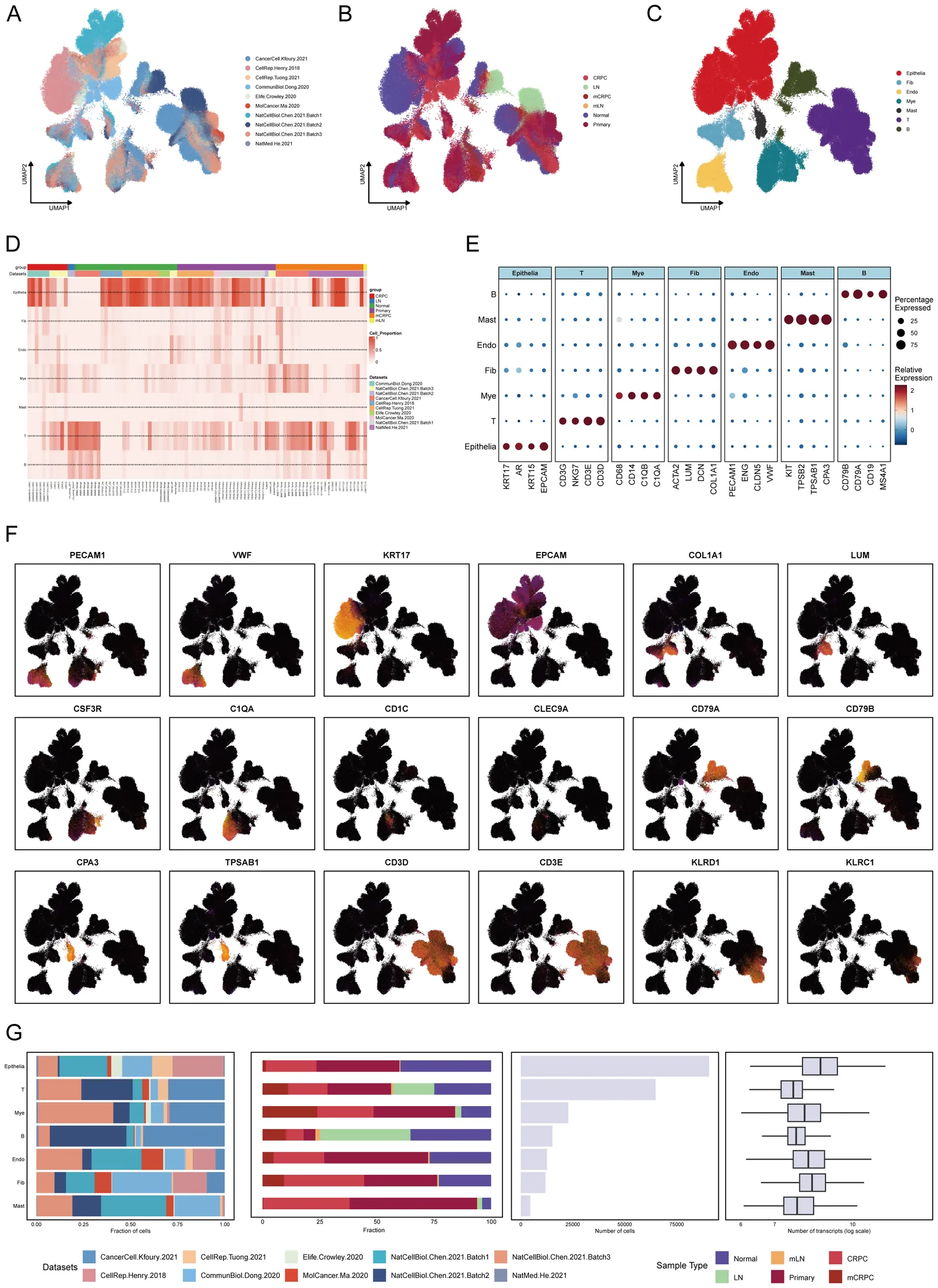
Panoramic molecular and cellular landscape of prostate cancer derived from a diverse patient cohort via scRNA-seq. **(A)** UMAP plot shows 222,529 single cells integrated from 10 independent studies, colored by data source. **(B)** UMAP visualization of tumor subtype heterogeneity, with single cells colored according to distinct molecular subtypes. **(C)** Major cell populations were annotated using established marker genes and include B cells, endothelial cells, epithelial cells, fibroblasts, mast cells, myeloid cells, T cells, and B cells. **(D)** A heatmap displays the relative abundance of each major cell type across all samples within the combined dataset. **(E)** A dot plot illustrates canonical marker expression across the seven principal cellular lineages, where dot size indicates the fraction of expressing cells and color represents average expression intensity. **(F)** Representative marker gene expression patterns employed for cell-type classification are shown, encompassing: epithelial (KRT17, EPCAM), fibroblast (COL1A1, LUM); myeloid subsets including neutrophils (CSF3R), macrophages (C1QA), cDC2 (CD1C), and cDC1 (CLEC9A); B cells (CD79A, CD79B); mast cells (CPA3, TPSAB1); T cells (CD3D, CD3E, KLRD1, KLRC1); and endothelial cells (PECAM1, VWF). **(G)** Analytical summaries are presented, detailing the dataset source of each cell population (left), tissue-type distribution per cell category (middle left), total cellular counts (middle right), and sequencing depth (right), together confirming balanced integration and consistent transcriptional coverage.

To systematically characterize the cellular ecosystem of prostate cancer, we integrated 222,529 single-cell transcriptomes from 10 independent studies spanning normal tissue, primary tumors, castration-resistant prostate cancer (CRPC), and metastatic CRPC (mCRPC) (**Figures 1A**; **Supplementary Figures 1B**, **C**). Following quality control and Harmony-based batch correction, cells from different datasets aligned closely in a shared UMAP space, indicating effective removal of technical variations across cohorts. Pre-correction UMAP revealed dataset-driven clustering, whereas post-correction UMAP showed intermingling of cells from different studies within shared cell-type clusters, supporting successful batch integration.

UMAP visualization revealed substantial inter- and intra-tumoral heterogeneity, with malignant cells partitioning into multiple transcriptional states corresponding to distinct molecular subtypes (**Figure 1B**). Based on canonical lineage markers, all cells were annotated into major compartments including epithelial/tumor cells, fibroblasts, myeloid cells, T/NK cells, B cells, endothelial cells, mast cells, and proliferating cells (**Figures 1C**; **Supplementary Figure 1A**). The relative abundance of each lineage varied markedly across individual samples (**Figures 1D**; **Supplementary Figures 1D**, **E**), reflecting pronounced compositional diversity among patients and disease stages. Dot-plot analysis confirmed robust and specific expression of canonical markers across lineages: epithelial cells expressed EPCAM and KRT17; fibroblasts expressed COL1A1 and LUM; macrophages expressed C1QA; neutrophils expressed CSF3R; dendritic cells expressed CD1C or CLEC9A; T/NK cells expressed CD3D, CD3E and KLRD1; B cells expressed CD79A/B; mast cells expressed CPA3 and TPSAB1; and endothelial cells expressed PECAM1 and VWF (**Figures 1E, F**).

Assessment of dataset origin, tissue source, cell counts, and sequencing depth demonstrated balanced representation across lineages and cohorts, supporting the robustness of downstream comparative analyses (**Figure 1G**). Collectively, this integrated atlas establishes a high-resolution cellular framework for dissecting stromal and immune remodeling throughout prostate cancer progression.

### 3.2 POSTN^+^ CAFs define a profibrotic fibroblast program enriched in advanced prostate cancer

**Figure 2:**
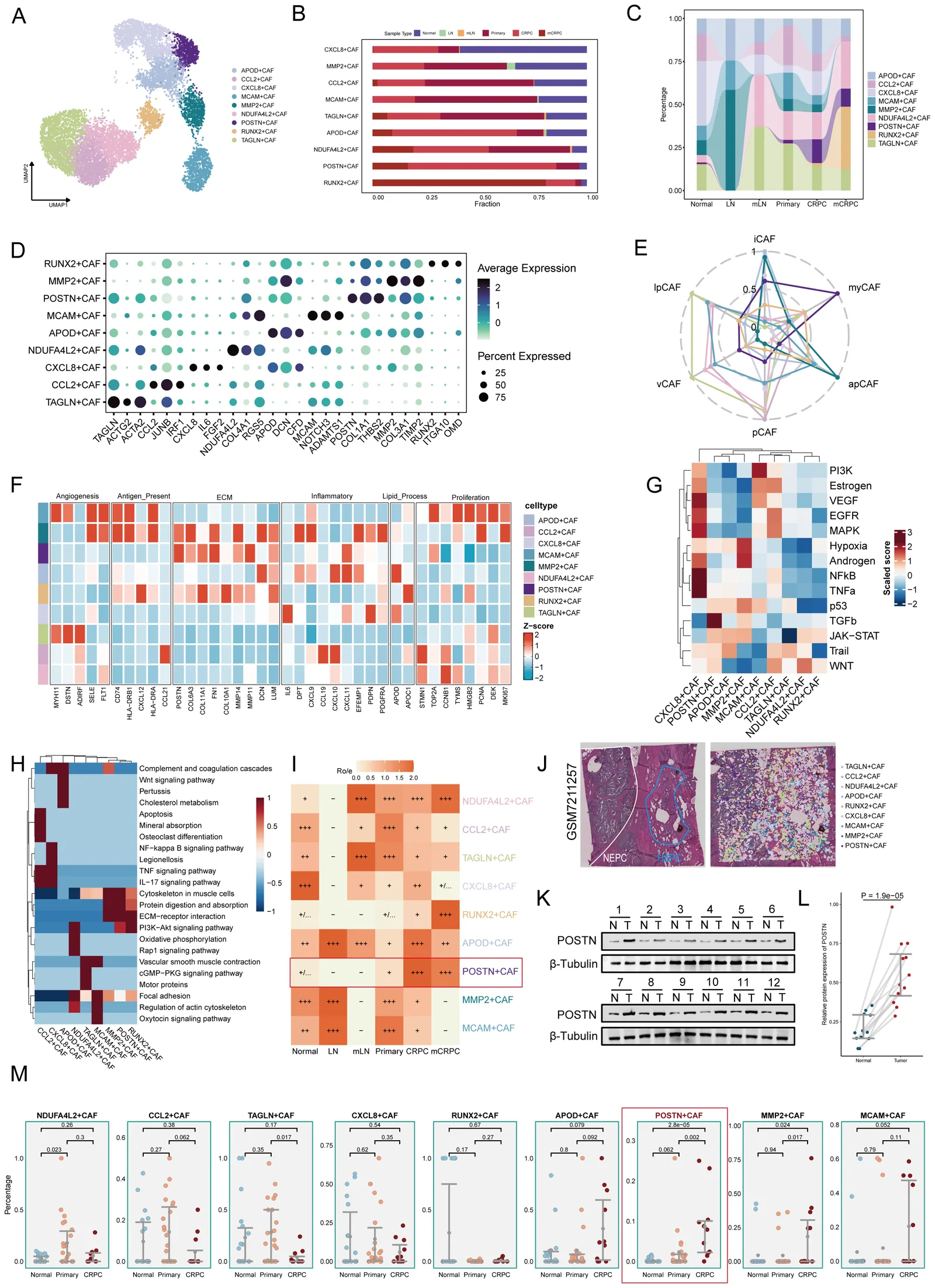
Comprehensive analysis of CAF heterogeneity and plasticity in prostate cancer. **(A)** UMAP visualization showing nine transcriptionally distinct CAF subsets (APOD^+^, CCL2^+^, CXCL8^+^, MCAM^+^, MMP2^+^, NDUFA4L2^+^, POSTN^+^, RUNX2^+^, and TAGLN^+^ CAFs) identified from integrated single-cell data. **(B)** The proportional contribution of tissue sources to each of the nine CAF subsets, demonstrating a specific enrichment of POSTN^+^CAFs in advanced (CRPC/mCRPC) disease. **(C)** Stacked bar plot displays the relative abundance of cancer-associated fibroblast (CAF) subsets across six tissue types, color-coded by subpopulation. **(D)** A dot plot visualizes signature marker expression across CAF subclusters, with dot size indicating the proportion of positive cells and color representing average normalized expression. **(E)** A radar chart illustrates the mean enrichment score, derived via AUCell, for each of the six established CAF signatures across the newly classified CAF subtypes. **(F)** Pathway activity levels across CAF subtypes are depicted in a heatmap, revealing elevated programs related to extracellular matrix organization, collagen production, and cellular contractility within POSTN^+^ CAFs. **(G)** Comparative signaling pathway activity, inferred using PROGENy, highlights pronounced TGF-β pathway activation specifically in POSTN^+^ CAF populations. **(H)** Enriched KEGG pathways for the differentially expressed genes characteristic of each CAF subtype are displayed, where color intensity corresponds to the scaled –log10-transformed adjusted p-value. **(I)** Tissue preference of each CAF subtypes estimated by Ro/e score. +++, Ro/e > 1; ++, 0.8 < Ro/e ≤ 1; +, 0.2 ≤ Ro/e ≤ 0.8; ±, 0 < Ro/e < 0.2; −, Ro/e = 0. **(J)** Spatial mapping of histology and cell types: a histopathological image of the tissue section (top) and the spatial distribution of fibroblast subtypes (bottom), deconvoluted by CellTrek from spatial transcriptome data. **(K)** Western blot analysis shows elevated POSTN protein expression in tumor compared to normal tissues from the in-house cohort. Note: bulk tissue analysis does not distinguish cellular sources; CAF-specific expression is inferred from scRNA-seq data. **(L)** Scatter plot showing paired POSTN protein expression levels in tumor and control samples (paired t-test). Each point represents one patient sample (n = 12 pairs). **(M)** Scatterplots depicting the proportions of fibroblast subtypes infiltration in Normal, Primary, and CRPC samples.

To resolve stromal heterogeneity in prostate cancer, fibroblasts were extracted from the integrated atlas and subjected to unsupervised reclustering (S2A, S2B). This analysis identified nine transcriptionally distinct cancer-associated fibroblast (CAF) subsets, annotated as APOD^+^, CCL2^+^, CXCL8^+^, MCAM^+^, MMP2^+^, NDUFA4L2^+^, POSTN^+^, RUNX2^+^, and TAGLN^+^ CAFs based on their marker-gene profiles (**Figure 2A**). It should be noted that these clusters represent transcriptional states rather than necessarily discrete, stable subtypes, and cells may transition between states. The distribution of CAF subsets varied markedly across tissue sources and disease stages (S2C). POSTN^+^ CAFs exhibited preferential expansion in CRPC and mCRPC samples compared with normal and primary tumors (**Figures 2B, C**), indicating a disease-stage-associated shift within the fibroblast compartment. Quantification of infiltration further demonstrated a progressive increase in POSTN^+^ CAF proportions from normal tissue to primary and advanced disease (**Figure 2M**; **Supplementary Figure 2D**).

Marker-gene analysis showed that POSTN^+^ CAFs were distinguished by high expression of core extracellular-matrix and stromal-remodeling genes, with POSTN, COL1A1, and THBS2 representing the most specific markers of this subset (**Figure 2D**). POSTN^+^ CAFs displayed a transcriptional profile consistent with an ECM-remodeling, myofibroblast-like (myCAF) state. In contrast, inflammatory CAF populations were enriched for chemokine programs (e.g., CCL2^+^ and CXCL8^+^), whereas MCAM^+^ CAFs displayed vascular-associated features. AUCell-based scoring revealed that POSTN^+^ CAFs exhibited the highest myofibroblast-like (myCAF) signature scores among all CAF subsets (**Figure 2E**), consistent with a contractile and matrix-producing phenotype.

Consistent with these transcriptional features, pathway-activity profiling indicated that POSTN^+^ CAFs showed elevated extracellular-matrix organization and collagen-associated programs relative to other CAF populations (**Figures 2F**; **Supplementary Figure 2E**). PROGENy analysis further demonstrated that TGF-β signaling activity reached its highest level in POSTN^+^ CAFs compared with other subsets (**Figure 2G**), supporting their association with activated and fibrotic stromal states.

KEGG pathway analysis of genes preferentially expressed in POSTN^+^ CAFs revealed enrichment in several key biological processes, including muscle cell cytoskeletal organization, protein metabolism and uptake, extracellular matrix-receptor crosstalk, and PI3K–Akt signaling (**Figure 2H**), reflecting coordinated regulation of matrix deposition, structural dynamics, and survival signaling.

Tissue-preference analysis using Ro/e scores demonstrated that POSTN^+^ CAFs were selectively enriched in advanced disease, showing a pronounced bias toward CRPC and mCRPC samples, whereas other CAF subsets exhibited broader or normal-associated distributions (**Figure 2I**). Integration with spatial transcriptomic data using CellTrek further revealed that POSTN^+^ CAFs localized predominantly within tumor-adjacent stromal regions and were closely apposed to malignant epithelial compartments (**Figure 2J**).

To validate the elevated expression of POSTN at the protein level, we performed Western blotting on paired clinical samples. Notably, POSTN protein levels were consistently higher in tumor tissues compared to adjacent normal tissues (**Figure 2K**). Quantitative densitometry further confirmed a significant upregulation of POSTN in tumor samples (**Figure 2L**, P < 0.001). It is important to note that Western blot analysis of bulk tissue cannot distinguish cellular sources of POSTN expression; therefore, these results validate elevated POSTN protein in the tumor microenvironment but do not prove CAF-specific expression. CAF-specific POSTN expression is supported by our single-cell transcriptomic data. These results are consistent with our scRNA-seq identification of the POSTN^+^ CAF subpopulation.

### 3.3 Trajectory analysis reveals POSTN^+^ CAF-initiated differentiation and STAT1-centered regulatory programs in prostate cancer

**Figure 3:**
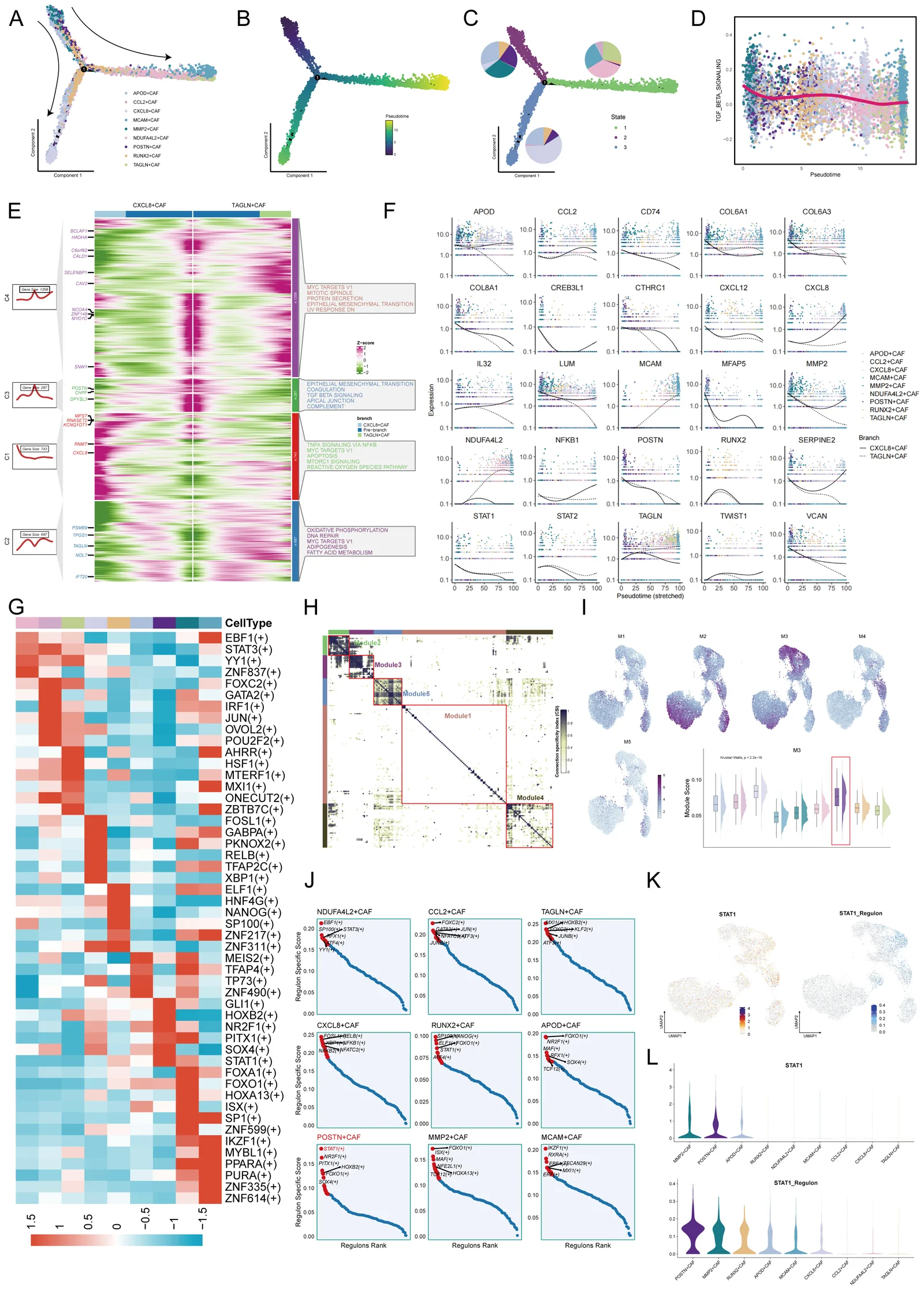
Trajectory analysis of cancer-associated fibroblast (CAF) subtypes in prostate cancer. **(A)** The developmental trajectory of CAFs was inferred by monocle 2 and colored according to the subtype.Each point represents a single cell **(B)** The pseudotime of the developmental trajectory. The colors from purple to yellow indicate values from low to high, respectively. Each point represents a single cell **(C)** The predicted cellular states along the trajectory. Each circle represents a state, with its colored segments showing the proportion of distinct CAF subtypes within that state.Each point represents a single cell **(D)** A dot plot displays the correlation between pseudotime progression and TGF-β signaling scores, with individual colors indicating specific CAF subtypes.Each point represents a single cell colored by CAF subtype **(E)** A heatmap illustrates the expression of genes significantly associated (q < 1e−3) with branching cell fates in CAFs (left). Box plots highlight the top five enriched hallmark pathways in each gene cluster derived from this analysis (right).Each column represents a gene; each row represents a cell. **(F)** Expression dynamics of selected genes along pseudotime demonstrate sequential activation of extracellular matrix-related factors (COL6A1, COL6A3, LUM, POSTN) and transcriptional regulators (STAT1, STAT2, VCAN) associated with fibroblast state transitions.Expression dynamics plotted per cell along pseudotime. **(G)** A heatmap presents the average activity of the most differentially active transcriptional regulons in each CAF subtype, as inferred from pySCENIC analysis. Each point or bar represents a single cell or CAF subtype, respectively. **(H)** Analysis of transcription factor regulatory modules across CAF subpopulations.Each point or bar represents a single cell or CAF subtype, respectively. **(I)** FeaturePlot visualization of regulon module activity across CAF subtypes, accompanied by a violin plot that contrasts the activity of the M3 regulon module among these subtypes. Each point or bar represents a single cell or CAF subtype, respectively. **(J)** Dot plots rank the six most specifically activated transcription factors in each CAF subtype based on their regulon specificity scores. Each point or bar represents a single cell or CAF subtype, respectively. **(K)** UMAP projections illustrate STAT1 expression (left) and its corresponding regulon activity (right) within fibroblast subpopulations, revealing clear enrichment in POSTN^+^ CAF clusters. Each point or bar represents a single cell or CAF subtype, respectively. **(L)** Violin plots compare STAT1 expression levels (top) and STAT1 regulon activity (bottom) across CAF subsets, with the highest activity observed in POSTN^+^ CAFs, supporting its role as a key transcriptional driver associated with fibroblast state transitions and ECM remodeling. Each point or bar represents a single cell or CAF subtype, respectively.

To delineate the dynamic evolution of cancer-associated fibroblasts (CAFs) in prostate cancer, we reconstructed CAF developmental trajectories using Monocle2 (S3A, S3B). The inferred trajectory organized CAF subtypes along a continuous pseudotime axis based on transcriptional similarity (**Figures 3A, B**). Notably, POSTN^+^ CAFs were predominantly located at the root of the trajectory, suggesting that this population represents a transcriptionally early state. However, we acknowledge that pseudotime inference alone cannot establish true developmental lineage or prove that POSTN^+^ CAFs directly differentiate into other CAF states.

State inference further revealed structured transitions along the developmental path, with distinct CAF compositions across trajectory states (**Figure 3C**). The distribution of CAF subtypes varied across states, supporting progressive specialization during fibroblast evolution. Correlation analysis indicated that pseudotime progression was associated with changes in TGF-β signaling activity across CAF subsets (**Figure 3D**; **Supplementary Figures 3C**, **D**). To investigate fate decisions along the trajectory, we performed branch-expression analysis, which identified significant gene modules associated with CAF fate bifurcation (q < 1×10^-^³) (**Figure 3E**). These gene clusters were enriched in hallmark pathways related to extracellular-matrix (ECM) organization, inflammatory signaling, cytoskeletal regulation, and transcriptional control, highlighting extensive functional reprogramming during CAF differentiation.

Dynamic profiling across pseudotime showed coordinated expression changes in ECM components (COL6A1, COL6A3, LUM, POSTN) and regulators (STAT1, STAT2, VCAN) (**Figure 3F**), indicating concurrent matrix and transcriptional remodeling during CAF state transitions.

Importantly, the trajectory structure positioned POSTN^+^ CAFs at the developmental origin and revealed two distinct differentiation branches. One branch preferentially gave rise to CXCL8^+^ CAFs, corresponding to an inflammatory-associated fate, whereas the other branch was enriched for TAGLN^+^ CAFs, representing a contractile and cytoskeleton-related phenotype. This bifurcation is consistent with the interpretation that POSTN^+^ CAFs may represent a progenitor-like fibroblast state from which functionally specialized CAF states could potentially emerge through transcriptional reprogramming.

To further characterize transcriptional regulation during CAF differentiation, we inferred regulon activity using pySCENIC. Differential-regulon analysis revealed distinct regulatory landscapes across CAF subtypes (**Figure 3G**). Regulatory-module analysis organized transcription factors into functional modules governing CAF identity (**Figure 3H**), among which a specific module showed preferential activation in POSTN^+^ CAFs (**Figure 3I**). Ranking of subtype-specific transcription factors demonstrated that STAT1 was among the most prominent regulators associated with POSTN^+^ CAFs (**Figure 3J**; **Supplementary Figure 3E**). Consistently, UMAP visualization showed enrichment of STAT1 expression and regulon activity within POSTN^+^ CAF clusters (**Figure 3K**), which was further confirmed by quantitative comparison across CAF subsets (**Figure 3L**). These results identify STAT1-centered transcriptional programs as a core feature of POSTN^+^ CAF biology. We emphasize that pseudotime inference provides a model of transcriptional state transitions rather than definitive evidence of lineage differentiation, and the proposed progenitor-like role of POSTN^+^ CAFs requires experimental validation using lineage-tracing approaches.

### 3.4 POSTN^+^ CAFs promote an immune-excluded niche through extracellular matrix remodeling in prostate cancer

**Figure 4:**
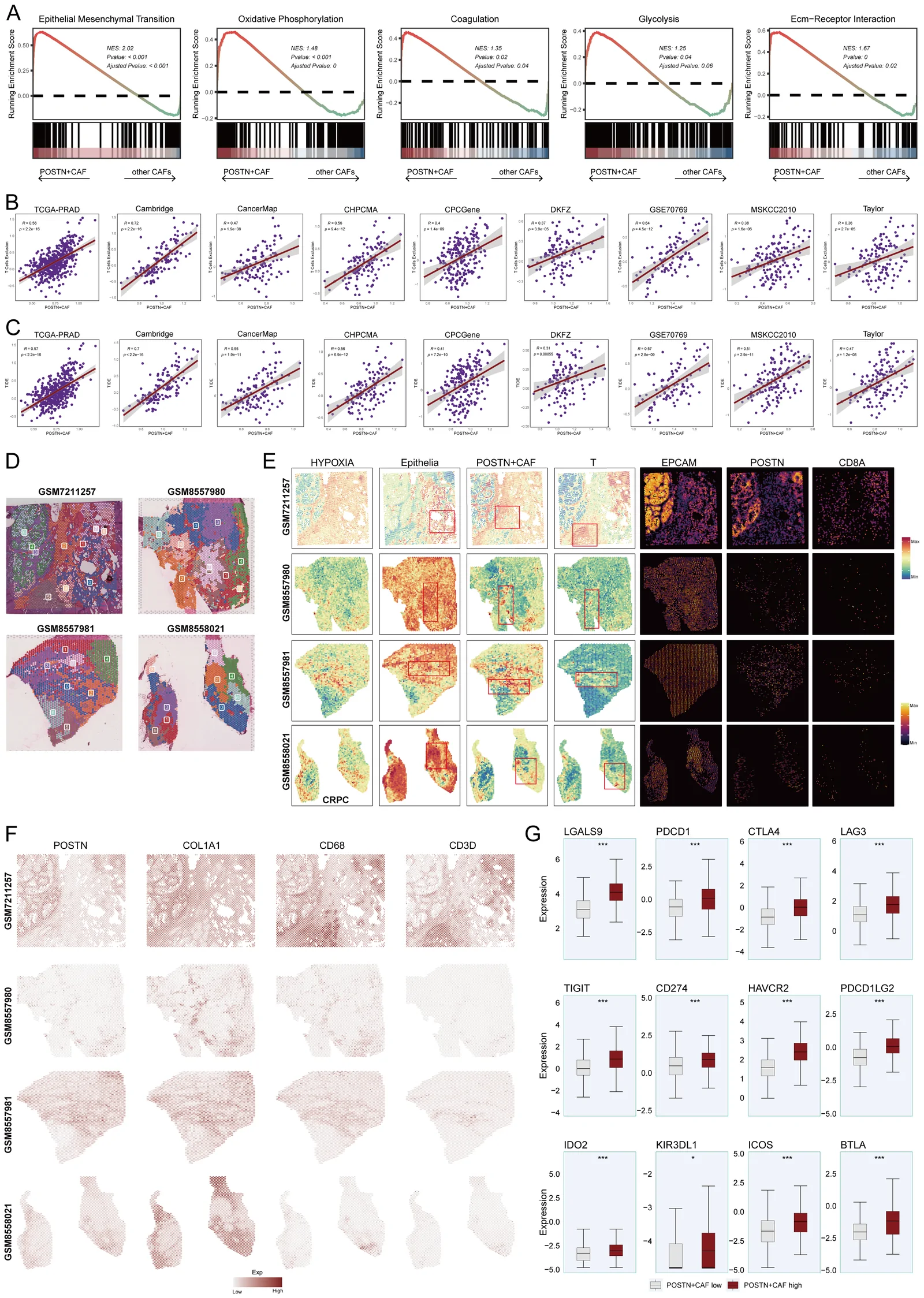
POSTN^+^CAFs establish an ECM-remodeled, immune-restrictive microenvironment in prostate cancer. **(A)** Gene set enrichment analysis (GSEA) of the Epithelial Mesenchymal Transition, Oxidative Phosphorylation, Coagulation, Glycolysis and ECM-Receptor Interaction pathways between POSTN+CAFs and other CAFs. The genes were ranked by fold change in expression between these two conditions. **(B)** Correlation analysis between POSTN^+^ CAF abundance and T-cell exclusion scores across 9 independent bulk transcriptomic cohorts. Each point represents one patient cohort (n = 9 independent cohorts). (all p < 1×10^-5^) **(C)** Correlation analysis between POSTN^+^ CAF abundance and Tide scores across 9 independent bulk transcriptomic cohorts. A strong and consistent positive correlation was observed (all p < 1×10^-5^), supporting the role of POSTN^+^ CAFs in promoting an immune-excluded tumor microenvironment. Each point represents one patient cohort (n = 9 independent cohorts). **(D)** Tissue architecture mapping of four PRAD samples via unsupervised clustering of spatial transcriptomic data. Each spot represents one spatial transcriptomic spot. **(E)** SpatialFeaturePlot visualization of hypoxia, malignant cells, POSTN^+^ CAFs, and T-cell signatures, along with spatial expression maps of EPCAM, POSTN, and CD8A, across four spatial transcriptomic tissue sections. Each spot represents one spatial transcriptomic spot. **(F)** Enhanced spatial feature plots showing the expression of POSTN, CD3D, CD68, and COL1A1 in tissue sections, showing co-localization of POSTN and COL1A1 with macrophage-enriched (CD68^+^) but T-cell–depleted (CD3D^+^) regions, consistent with an immune-excluded stromal niche. Each spot represents one spatial transcriptomic spot. **(G)** Boxplots comparing the expression of immune checkpoint molecules between POSTN^+^ CAF–high and –low samples in TCGA cohort. Each point represents one patient sample from the TCGA-PRAD cohort. *p < 0.05,**p < 0.01,***p <0.001.

To investigate the functional impact of POSTN^+^ CAF accumulation within the prostate tumor microenvironment, we systematically analyzed their transcriptional programs, immune associations, and spatial organization. Gene-set-enrichment analysis comparing POSTN^+^ CAFs with other CAF subtypes revealed significant enrichment of pathways related to epithelial–mesenchymal transition, ECM–receptor interaction, glycolysis, coagulation, and oxidative phosphorylation (**Figure 4A**). These results indicate that POSTN^+^ CAFs display an active matrix-remodeling and metabolically engaged phenotype.

We next examined the relationship between POSTN^+^ CAF abundance and tumor immune contexture using bulk transcriptomic datasets. Across nine independent cohorts, higher POSTN^+^ CAF levels were consistently associated with increased T-cell exclusion scores (all p < 1×10^-5^) (**Figure 4B**), indicating reduced lymphocyte infiltration in POSTN^+^ CAF-enriched tumors. In parallel, POSTN^+^ CAF abundance showed a strong positive correlation with TIDE scores across the same cohorts (all p < 1×10^-5^) (**Figure 4C**), linking this fibroblast population to immune-evasion-associated transcriptional programs.

To validate these associations at the tissue level, we analyzed spatial transcriptomic data from prostate cancer samples. Unsupervised clustering delineated distinct tissue-architecture patterns across sections (**Figure 4D**). Spatial-feature mapping revealed that POSTN^+^ CAF signatures were preferentially localized to malignant and hypoxic regions, whereas T-cell signatures exhibited limited overlap with these stromal compartments (**Figure 4E**). Expression maps of EPCAM, POSTN, and CD8A further illustrated spatial separation between POSTN-enriched stroma and cytotoxic T-cell infiltration.

Spatial transcriptomic visualization revealed that POSTN and COL1A1 expression were enriched in regions showing macrophage-associated transcriptional signatures, while showing reduced overlap with T-cell-associated areas. This spatial organization is consistent with a model in which POSTN^+^ CAFs may participate in forming ECM-dense stromal zones, although direct validation at single-cell resolution is warranted. This spatial organization indicates that POSTN^+^ CAFs participate in forming ECM-dense stromal zones that are permissive for myeloid accumulation but restrictive for T-cell penetration.

Finally, immune-checkpoint gene expression profiles were examined within the TCGA prostate cancer dataset, categorized by levels of POSTN^+^ CAF presence. Tumors characterized by abundant POSTN^+^ CAFs displayed markedly different expression patterns across several immune-checkpoint molecules relative to those with low POSTN^+^ CAF levels (**Figure 4G**), reinforcing the connection between POSTN^+^ CAF enrichment and an immunosuppressive tumor environment.

Together, these results indicate that POSTN^+^ CAFs contribute to extracellular matrix reorganization, altered metabolic states, immune exclusion, and unique spatial organization in prostate cancer, underscoring their pivotal function in molding the tumor microenvironment.

### 3.5 Myeloid compartment analysis links POSTN^+^ CAFs to M2-like APOE^+^ macrophages

**Figure 5:**
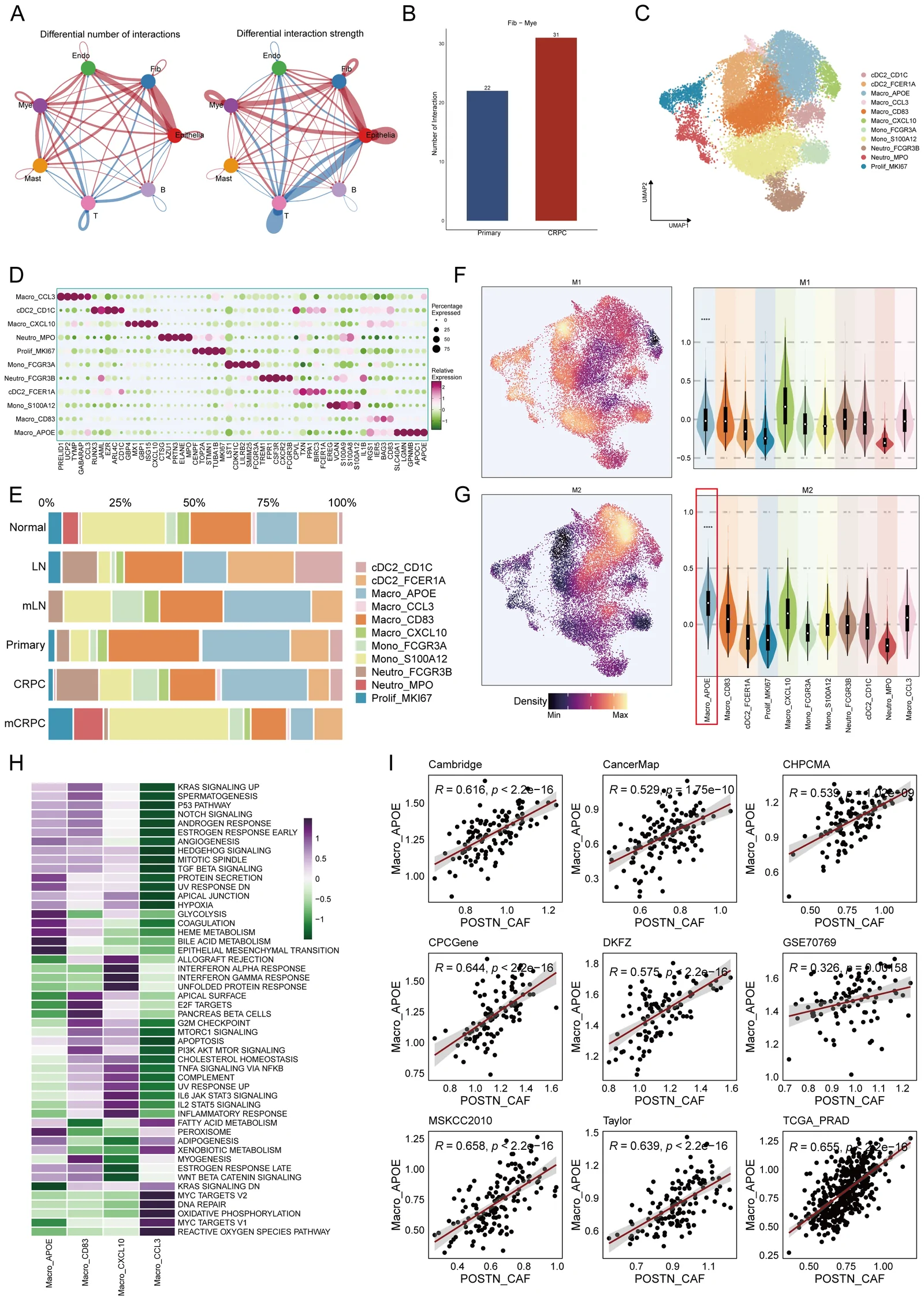
Comprehensive profiling of myeloid heterogeneity identifies M2-like, immunoregulatory APOE^+^ macrophages as the subset most strongly correlated with POSTN^+^ CAFs in prostate cancer. **(A)** Circle plots showing differential interaction numbers and strengths between Primary and CRPC samples in the integrated cohort. Blue lines indicate decreased communication, while red lines indicate increased communication in CRPC compared to Primary samples. Interaction networks are computed at the cell-population level. **(C–E)** Each point represents a single cell. **(B)** Bar plot quantification of fibroblast–myeloid cell–cell interactions shows a significant increase in CRPC compared to Primary tumors (31 vs. 22 total interactions). Interaction networks are computed at the cell-population level. **(C–E)** Each point represents a single cell. **(C)** Uniform manifold approximation and projection (UMAP) plot of individual myeloid cells. Each dot denotes one cell; the color represents the myeloid subtype. **(D)** Dotplot displaying the top three marker genes across myeloid cell subsets. **(E)** Barplot showing the proportions of each myeloid subset across six tissue sources. **(F)** UMAP-based density plot of M1 macrophage gene signature scores (left), accompanied by a corresponding violin plot comparing M1 scores across cell populations (right). Each point represents a single cell; violin plots show per-cell signature scores. **(G)** UMAP-based density plot of M2 macrophage gene signature scores (left), accompanied by a corresponding violin plot comparing M2 scores across cell populations (right). Each point represents a single cell; violin plots show per-cell signature scores. **(H)** Heatmaps illustrate the activity intensity of macrophage subtypes in hallmark pathways using GSVA analysis, with z-score normalization (range: −2 to 2). Each column represents a pathway; each row represents a myeloid subtype. **(I)** Scatter plots showing the correlation between the score of POSTN^+^ CAF and APOE^+^ macrophage across 9 independent PRAD datasets. Each point represents one independent PRAD dataset (n = 9 cohorts).

To characterize myeloid heterogeneity and its association with fibroblast remodeling in prostate cancer, we first compared intercellular communication patterns between primary and CRPC samples. Circle plots revealed widespread remodeling of cell–cell interactions in CRPC, with increased interaction numbers and strengths relative to primary tumors (**Figure 5A**). Quantification of fibroblast–myeloid interactions showed a marked increase in CRPC compared with primary samples (31 vs. 22 interactions), indicating enhanced stromal–myeloid crosstalk during disease progression (**Figure 5B**).

We next resolved the myeloid compartment at single-cell resolution. We refer to APOE^+^ macrophages as ‘M2-like/immunoregulatory’ to reflect their transcriptional features, which include elevated M2-associated gene expression, enrichment of lipid metabolism pathways, and immunoregulatory programs, rather than implying a fixed polarization state. UMAP projection separated myeloid cells into distinct subsets, including monocytes, dendritic cells, and multiple macrophage populations (**Figure 5C**). To validate cell-type annotation, we examined the top marker genes for each myeloid subset. Dot-plot visualization showed specific and robust expression of canonical markers across clusters, confirming the accuracy of myeloid cell classification (**Figure 5D**; **Supplementary Figures S4A, B**). The distribution of myeloid subsets varied across tissue sources and disease stages, with macrophage populations showing increased representation in advanced disease samples (**Figure 5E**).

To assess macrophage polarization, we calculated M1 and M2 signature scores. Density and violin plots showed that M1 scores were relatively low across most macrophage populations (S4C), whereas M2 scores were selectively elevated in the APOE^+^ macrophage subset (**Figures 5F, G**; **Supplementary Figure 4D**). GSVA further demonstrated that APOE^+^ macrophages exhibited increased activity in hallmark pathways related to lipid metabolism, hypoxia response, and immunoregulatory programs compared with other myeloid subsets (**Figure 5H**), consistent with an M2-like, immunoregulatory transcriptional state with lipid-associated features.

We then examined the relationship between fibroblast and myeloid populations at the cohort level. Across nine independent PRAD datasets, POSTN^+^ CAF scores were positively correlated with APOE^+^ macrophage abundance (**Figure 5I**). This association was consistent across cohorts, indicating that tumors enriched in POSTN^+^ CAFs also display increased infiltration of M2-polarized APOE^+^ macrophages. Multiplex immunofluorescence staining (**Supplementary Figure 12**) showed that, compared with normal prostate tissue, POSTN fluorescence signal was markedly increased in tumor tissue, predominantly distributed in the periglandular stromal region in a network-like pattern, whereas POSTN signal was minimal in normal tissue. The density of CD68^+^ cells was generally higher in tumor tissue than in normal tissue, with a subset of CD68^+^ signal spatially overlapping with POSTN^+^ stromal regions. CD8^+^ cells were widely observed in normal tissue but were reduced in number in tumor tissue, where they were predominantly localized outside the POSTN^+^ stromal region. The merged images revealed distinct spatial distribution patterns of POSTN, CD68, and CD8 signals in tumor tissue.

### 3.6 MDK–NCL signaling is associated with preferential communication between POSTN^+^ CAFs and APOE^+^ macrophages

**Figure 6:**
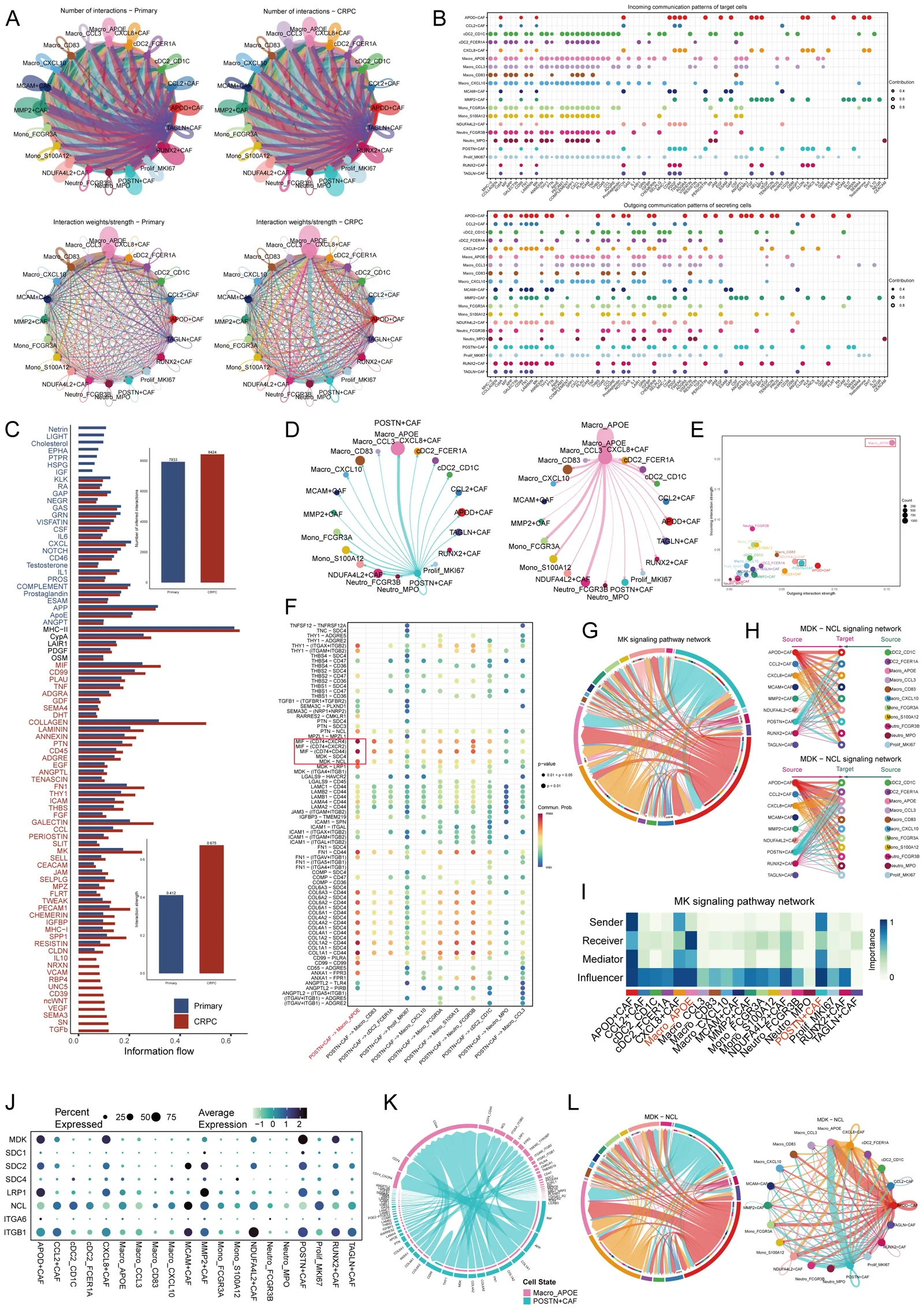
POSTN+ fibroblasts interact with APOE+ macrophages via the MDK-NCL axis. **(A)** Circos plots comparing the number and strength of ligand-receptor interactions between fibroblast and myeloid cell subclusters in primary versus CRPC samples. Interaction networks are computed at the cell-population level. **(B)** Bubble plots depicting the incoming communication patterns for target cell types (top) and the outgoing communication patterns for source/secretory cell types (bottom). Each bubble represents one ligand-receptor pair between defined cell populations. **(C)** Comparison of pathway activity strength and interaction numbers/strength between CRPC and primary tissue samples. Pathway-level analyses are computed across cell-type pairs. **(D)** Circos plots showing the number of interactions involving POSTN+ CAFs (left) and APOE+ macrophages (APOE+ Macro, right) with other cell types. Interaction networks are computed at the cell-population level. **(E)** Two-dimensional scatter plot of signaling role analysis for CAF and epithelial subclusters, indicating POSTN+ CAFs as dominant signal senders and APOE+ macrophages as the top signal receivers. Each point represents one CAF or epithelial subcluster. **(F)** Bubble plot displaying specific ligand-receptor pairs from POSTN+ CAFs to other myeloid cell types. Each bubble represents one ligand-receptor pair between defined cell populations. **(G)** Interaction network of all cell types within the MK signaling pathway. Pathway-level analyses are computed across cell-type pairs. **(H)** Paracrine interaction network and centrality scores for all cells within the MDK-NCL signaling pathway. Pathway-level analyses are computed across cell-type pairs. **(I)** Centrality scores for the interaction network in the MK signaling pathway. Pathway-level analyses are computed across cell-type pairs. **(J)** Bubble plot showing expression levels of ligands and receptors across different cell types within the MK signaling pathway. Each bubble represents one ligand-receptor pair between defined cell populations. **(K)** Chord diagram illustrating interactions between POSTN+ CAFs and APOE+ macrophages. Interaction networks are computed at the cell-population level. **(L)** Interaction network among all cell types within the MDK-NCL signaling pathway. Interaction networks are computed at the cell-population level.

Given the correlation between POSTN^+^ CAFs and APOE^+^ macrophages, we further investigated their predicted communication landscape using computational ligand-receptor inference. Comparison of ligand–receptor interactions between primary and CRPC samples showed a global increase in interaction number and strength between fibroblast and myeloid subclusters in CRPC (**Figure 6A**). Incoming and outgoing signaling analyses revealed enhanced signal reception by myeloid populations and increased signal emission from fibroblast subsets in advanced tumors (**Figure 6B**). Quantitative comparison of pathway activity and interaction strength revealed that multiple signaling pathways were globally enhanced in CRPC compared with primary tumors (**Figure 6C**).

Circos visualization of the cell–cell communication network showed that POSTN^+^ CAFs displayed extensive outgoing interactions with multiple surrounding cell types, and APOE^+^ macrophages also engaged broadly with other populations. Notably, the interaction links between POSTN^+^ CAFs and APOE^+^ macrophages were among the most prominent connections in the network, indicating preferential stromal–myeloid crosstalk in advanced disease (**Figure 6D**). Two-dimensional signaling-role analysis further positioned POSTN^+^ CAFs toward the sender-dominant region and APOE^+^ macrophages toward the receiver-dominant region, supporting a directional communication pattern from fibroblasts to macrophages (**Figure 6E**).

To dissect specific ligand–receptor interactions, we examined signaling from POSTN^+^ CAFs to myeloid populations. Bubble plots revealed that POSTN^+^ CAFs were predicted to preferentially communicate with APOE^+^ macrophages through defined ligand–receptor axes, with MDK–NCL representing the most prominent predicted interaction. In addition, immune-modulatory pairs such as MIF–(CD74+CD44) and MIF–(CD74+CXCR4) were also enriched, indicating coordinated regulation of macrophage behavior by stromal signals (**Figure 6F**). Pathway-level analysis highlighted enrichment of the MK (midkine) signaling pathway within the communication network (**Figure 6G**). Centrality analysis further showed that POSTN^+^ CAFs and APOE^+^ macrophages occupied high-centrality positions within the MK network (**Figures 6H, I**), indicating that these two populations serve as core hubs of MK-mediated stromal–myeloid communication.

Within this pathway, expression mapping showed that POSTN^+^ CAFs preferentially expressed the ligand MDK, whereas APOE^+^ macrophages showed higher expression of the receptor NCL (**Figure 6J**). Chord diagrams further illustrated dense interactions between POSTN^+^ CAFs and APOE^+^ macrophages (**Figure 6K**). Network reconstruction of the MDK–NCL axis predicted strong paracrine connections between these two populations relative to other cell types (**Figure 6L**).

### 3.7 Co-enrichment of POSTN^+^ CAFs and APOE^+^ macrophages associates with poor outcome and reduced immunotherapy benefit

**Figure 7:**
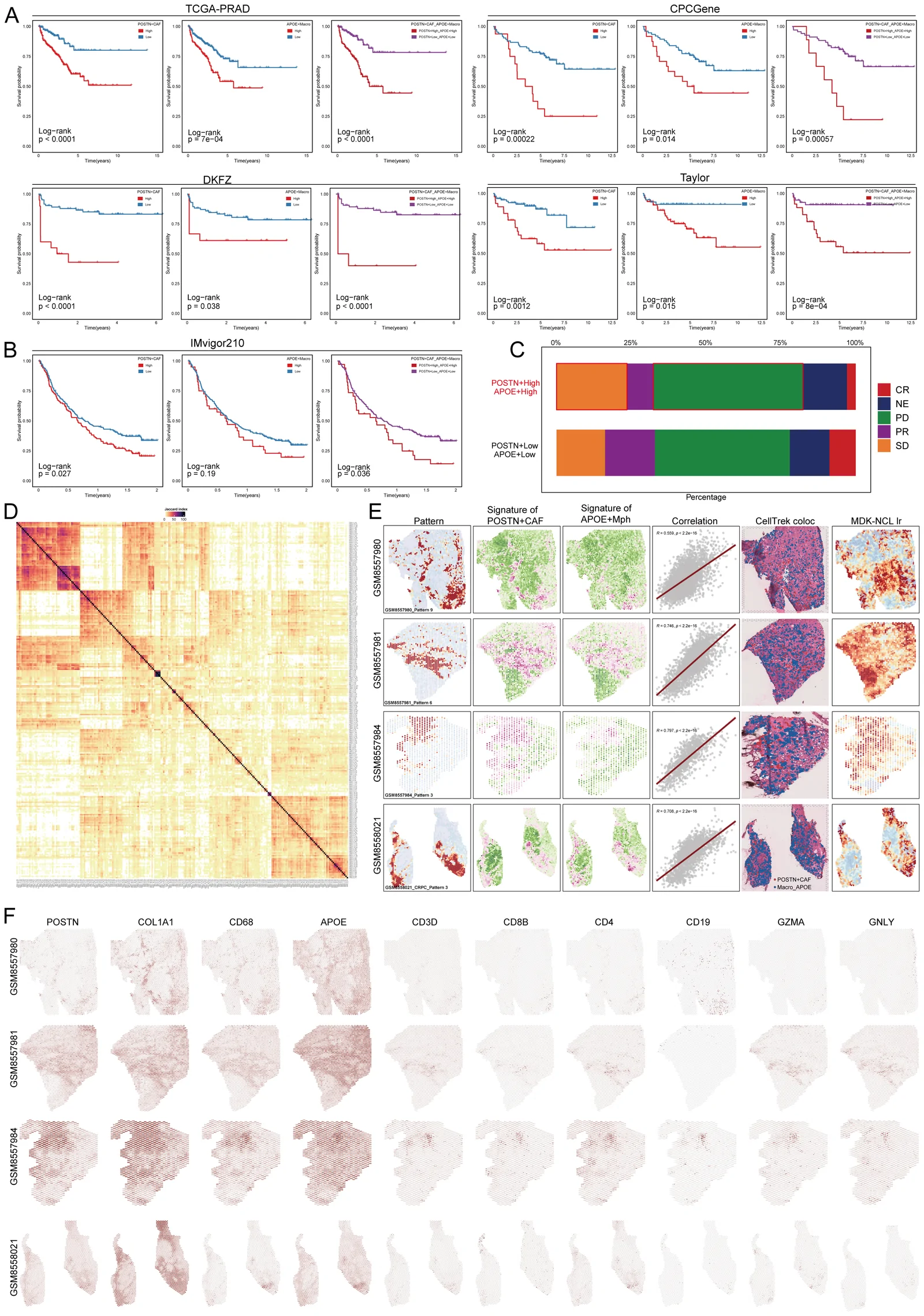
Co-enrichment of POSTN^+^ CAFs and APOE^+^ macrophages predicts adverse clinical outcomes and limited immunotherapy benefit. **(A)** Kaplan-Meier survival estimates across four transcriptomic cohorts (TCGA-PRAD, CPC-GENE, DKFZ, Taylor), comparing patients grouped by low versus high infiltration of: left, POSTN^+^ CAFs only; middle, APOE^+^ macrophages only; right, both cell types together. Each curve represents survival probability across patient groups; statistical comparisons are at the patient level. **(B)** Survival analysis in the IMvigor210 urothelial carcinoma immunotherapy cohort (cross-cancer exploratory analysis) stratifying patients by low and high infiltration of POSTN^+^ CAFs (left), APOE^+^ macrophages (middle), and their co-infiltration (right), visualized using Kaplan-Meier curves. Caution should be exercised in extrapolating these findings to prostate cancer. Each curve represents survival probability across patient groups; statistical comparisons are at the patient level. **(C)** Bar chart illustrating that patients exhibiting low combined infiltration of POSTN^+^ CAFs and APOE^+^ macrophages show a higher rate of complete/partial response (CR/PR), whereas stable/progressive disease (SD/PD) is more frequent in the high co-infiltration group. Proportions are calculated at the patient level within each response category. **(D)** Heatmap depicting pairwise similarity (Jaccard index) of spatially defined gene programs identified by SpaGene across samples, based on overlapping top-ranked genes. Each spot represents one spatial transcriptomic spot. **(E)** For four representative PRAD samples (left to right): Spatial mapping of POSTN^+^ CAF and APOE^+^ macrophage signature scores; dot plot indicating their significant positive spatial correlation; CellTrek-based reconstruction of their spatial distributions; and SpaGene mapping highlighting colocalization of the MDK–NCL ligand–receptor pair. Each spot represents one spatial transcriptomic spot. **(F)** High-resolution spatial expression patterns in four prostate cancer specimens, visualizing key markers: POSTN, COL1A1 (CAFs); CD68, APOE (macrophages); CD3D, CD8B, CD4, CD19 (lymphocytes); and GZMA, GNLY (cytotoxic activity).Each pixel represents expression intensity at the spot level.

To evaluate the clinical relevance of POSTN^+^ CAFs and APOE^+^ macrophages, we examined their associations with patient survival across multiple cohorts. Kaplan–Meier analysis in four independent bulk transcriptomic datasets (TCGA-PRAD, CPC-GENE, DKFZ, and Taylor) showed that patients with high infiltration of POSTN^+^ CAFs exhibited significantly worse overall survival compared with those with low infiltration (**Figure 7A**, left). A similar unfavorable survival pattern was observed for APOE^+^ macrophages (**Figure 7A**, middle). Notably, patients with concurrent high infiltration of both POSTN^+^ CAFs and APOE^+^ macrophages showed the poorest prognosis among all groups (**Figure 7A**, right), indicating additive stratification value.

To explore the potential relevance of this stromal–myeloid axis for immunotherapy response, we examined the IMvigor210 anti-PD-L1 cohort (urothelial cancer), which provides a well-characterized immunotherapy dataset with clinical response annotations. Although this cohort represents a different cancer type, it offers an opportunity to assess whether the POSTN^+^ CAF and APOE^+^ macrophage signatures, defined from prostate cancer scRNA-seq, are associated with immunotherapy outcomes in a cross-cancer context. This analysis is exploratory and hypothesis-generating, and findings should be interpreted with appropriate caution. In this cohort, high POSTN^+^ CAF infiltration alone was associated with reduced survival benefit following treatment (**Figure 7B**, left), and APOE^+^ macrophage enrichment showed a similar trend (**Figure 7B**, middle). Patients harboring tumors with simultaneous high POSTN^+^ CAF and APOE^+^ macrophage infiltration exhibited the lowest survival probability after immunotherapy (**Figure 7B**, right).

Consistent with survival patterns, response-category analysis revealed that tumors with low co-infiltration of POSTN^+^ CAFs and macrophage programs had a higher proportion of complete or partial responses (CR/PR), whereas stable or progressive disease (SD/PD) was enriched in the high co-infiltration group (**Figure 7C**). This distribution suggests that stromal–myeloid co-enrichment may be associated with reduced immunotherapeutic efficacy, although this finding is derived from a urothelial cancer cohort and requires validation in prostate cancer-specific immunotherapy datasets, which are currently limited.

To investigate spatial coordination, SpaGene analysis quantified gene-program overlap using the Jaccard index, revealing structured spatial gene-program similarity across samples (**Figure 7D**). In four PRAD spatial transcriptomic sections, SpatialFeaturePlot mapping showed overlapping enrichment of POSTN^+^ CAF and APOE^+^ macrophage signatures (**Figure 7E**, left; **Supplementary Figure S9A**). Dot-plot analysis confirmed significant positive spatial correlations between these two programs (**Figure 7E**, second panel). CellTrek-based deconvolution further localized both cell types to shared tissue regions (**Figure 7E**, third panel), while SpaGene mapping identified spatial co-localization of the MDK–NCL ligand–receptor axis (**Figure 7E**, right), supporting localized paracrine signaling *in situ*.

High-resolution spatial visualization further demonstrated that POSTN and COL1A1 signals were concentrated in CD68^+^ and APOE^+^ macrophage-rich regions, whereas lymphocyte markers including CD3D, CD8B, CD4, CD19, GZMA, and GNLY showed reduced presence in these areas (**Figures 7F**; **Supplementary Figure 9B**). These spatial patterns indicate that regions enriched for POSTN^+^ CAFs and APOE^+^ macrophages coincide with lymphocyte-depleted niches.

### 3.8 Exploratory pan-cancer analysis suggests conserved enrichment and prognostic associations of POSTN^+^ CAFs and APOE^+^ macrophages

**Figure 8:**
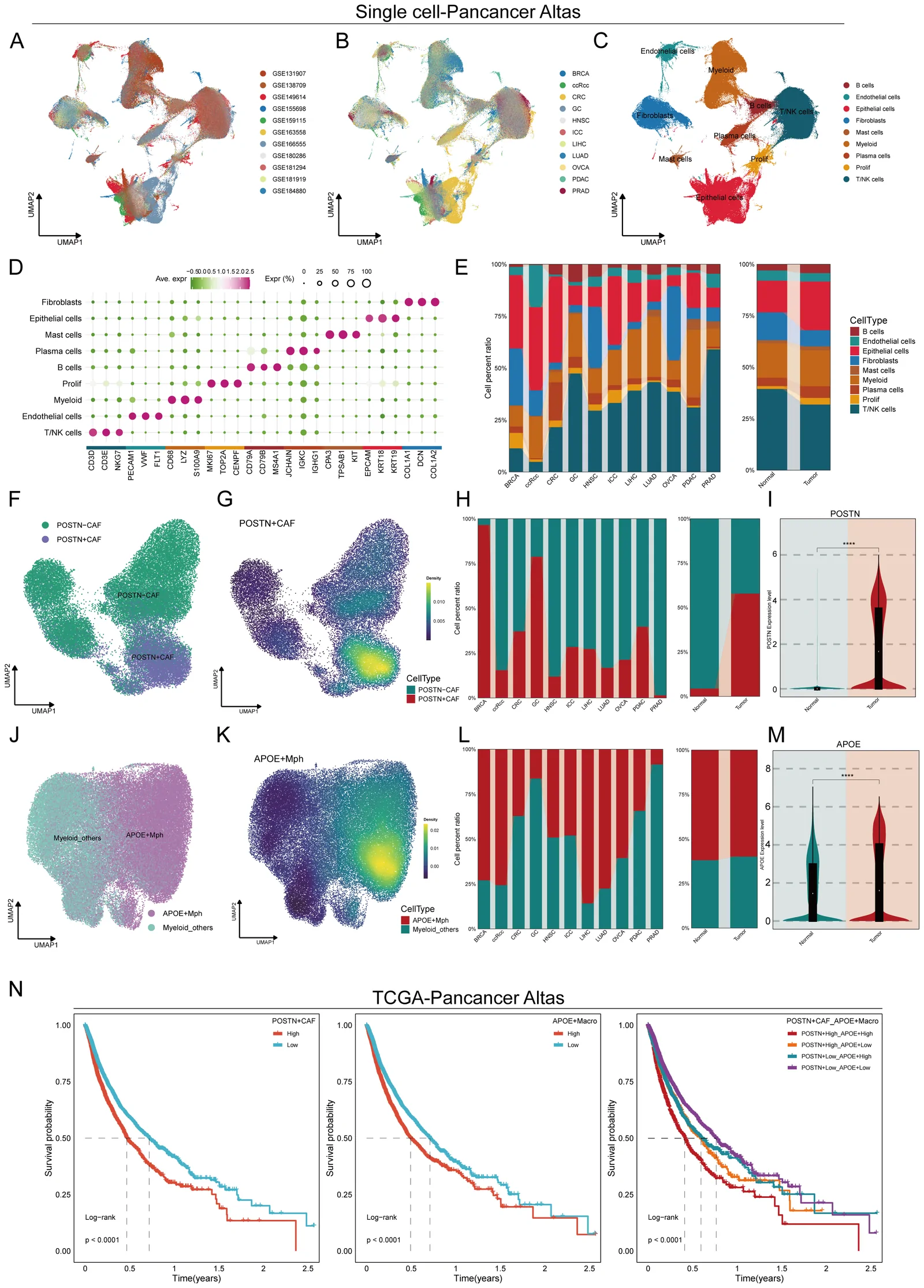
Characterization of POSTN+ CAF and APOE+ macrophages in pan-cancer cohort. **(A)** UMAP plot showing the integrated datasets. Each point represents a single cell, colored by its source dataset. Each point represents a single cell. **(B)** UMAP plot of the integrated tumor samples. Points (cells) are colored by cancer type. Each point represents a single cell. **(C)** UMAP plot showing nine major cell types. Points are colored by annotated cell identity. Each point represents a single cell. **(D)** Dot plot displaying the expression level and percentage of classical marker genes across the major cell types. Each point represents a single cell. **(E)** Bar plots showing the proportion of major cell types across 11 cancer types (left) and between matched tumor and adjacent normal tissues (right). Proportions are calculated at the sample level. **(F)** UMAP plot showing the distributions of POSTN^+^ CAFs and POSTN^-^ CAFs. Each point represents a single cell. **(G)** Density plot of the POSTN^+^ CAF signature score. Density distributions are computed per cell. **(H)** Bar plots showing the proportion of POSTN^+^ CAFs among all CAFs across 11 cancer types (left) and between tumor and adjacent normal tissues (right). Proportions are calculated at the sample level. **(I)** Violin plot showing the differential expression of POSTN between tumor and adjacent normal tissues. Each point represents one cell. **(J)** UMAP plot showing the distributions of APOE^+^ macrophages and APOE^-^ macrophages. Each point represents a single cell. **(K)** Density plot of the APOE^+^ macrophage signature score. Density distributions are computed per cell. **(L)** Bar plots showing the proportion of APOE^+^ macrophages among all myeloid cells across 11 cancer types (left) and between tumor and adjacent normal tissues (right). Proportions are calculated at the sample level. **(M)** Violin plot showing the differential expression of APOE between tumor and adjacent normal tissues. Each point represents one cell. **(N)** Kaplan-Meier survival analysis in the TCGA Pan-Cancer cohort. Patients were stratified by low versus high infiltration of: left, POSTN^+^ CAFs alone; center, APOE^+^ macrophages alone; right, both POSTN^+^ CAFs and APOE^+^ macrophages. Each curve represents survival probability across patients in the TCGA pan-cancer cohort.

To explore whether the POSTN^+^ CAF–APOE^+^ macrophage axis might extend beyond prostate cancer, we constructed a pan-cancer single-cell atlas integrating datasets from 11 cancer types. Given the tissue-specific nature of CAF and macrophage states, this analysis is exploratory and intended to generate hypotheses rather than establish definitive conservation. This section presents an exploratory, descriptive analysis intended to assess whether the POSTN^+^ CAF and APOE^+^ macrophage signatures identified in prostate cancer are observable in other tumor types. Given the descriptive nature of this analysis, we focus on reporting patterns of enrichment and spatial co-localization without inferring mechanistic conservation. UMAP visualization showed successful integration across datasets and tumor types (**Figures 8A, B**; **Supplementary Figures 10A–C, E–G**), with clear separation of major cellular compartments after annotation (**Figure 8C**). Dot-plot analysis confirmed canonical marker expression across epithelial, stromal, immune, and endothelial populations (**Figure 8D**). The proportional composition of major cell types varied across cancer types and between tumor and adjacent normal tissues (**Figure 8E**), indicating context-dependent microenvironment remodeling.

Within the fibroblast compartment, UMAP projection distinguished POSTN^+^ and POSTN^-^ CAF populations (**Figure 8F**). Density plots showed heterogeneous distribution of POSTN^+^ CAF signature scores across cells (**Figure 8G**). Quantification demonstrated that the proportion of POSTN^+^ CAFs among total CAFs varied across cancer types and was consistently higher in tumor tissues compared with matched adjacent normal samples (**Figures 8H**; **Supplementary Figure 10D**). Violin-plot analysis further showed elevated POSTN expression in tumors relative to normal tissues (**Figure 8I**).

Similarly, UMAP visualization separated APOE^+^ and APOE^-^ macrophage populations (**Figure 8J**). APOE^+^ macrophage signature scores displayed distinct density distributions (**Figure 8K**). Across cancer types, the fraction of APOE^+^ macrophages among myeloid cells were increased in tumors compared with adjacent normal tissues (**Figures 8L**; **Supplementary Figure 10H**), and APOE expression was significantly higher in malignant samples (**Figure 8M**).

Notably, spatial transcriptomic analysis across seven additional cancer types (BRCA, CESC, COAD, GBM, HNSC, LIHC, and KIRC) revealed that both POSTN and APOE expression were frequently enriched at the tumor–stroma boundary (interface regions) compared with core tumor or pure stromal areas (**Supplementary Figures 11A–G**). This conserved spatial co-localization at invasive fronts further underscores the pan-cancer relevance of the POSTN^+^ CAF–APOE^+^ macrophage axis in shaping the tumor microenvironment.

To explore clinical relevance at scale, Kaplan–Meier analysis in the TCGA pan-cancer cohort suggested that high infiltration of POSTN^+^ CAFs alone was associated with inferior survival (**Figure 8N**, left), and APOE^+^ macrophage enrichment showed a similar negative association (**Figure 8N**, middle). Patients with concurrent enrichment of both populations exhibited the most unfavorable survival outcomes (**Figure 8N**, right; **Supplementary Figure 10I**). While these findings are consistent across tumor types, they are exploratory and should be interpreted cautiously given the heterogeneity of CAF and macrophage states across tissues.

## 4 Discussion

This study delineates a spatially coordinated and functionally interdependent stromal-immune network that underlies disease progression and immune evasion in prostate cancer. By integrating single-cell and spatial transcriptomic profiles across the clinical continuum—from localized tumors to metastatic castration-resistant prostate cancer (mCRPC)—we have characterized a previously unrecognized POSTN^+^ CAF–APOE^+^ macrophage axis that is associated with a fibrotic, immune-excluded tumor microenvironment. Computational analyses suggest that POSTN^+^ CAFs represent a progenitor-like fibroblast state associated with a STAT1-dependent transcriptional program, which may transition toward specialized lineages and contribute to the desmoplastic stroma (21, 22). Inferred ligand-receptor analysis predicts privileged paracrine communication between these fibroblasts and M2-like APOE^+^ macrophages, predominantly via the MDK-NCL signaling pathway, thereby forming a spatially co-localized stromal-immune unit that physically excludes cytotoxic T-cell infiltration and correlates with unfavorable clinical outcomes and reduced response to immune checkpoint inhibition (23).

We emphasize that the proposed MDK-NCL signaling axis, while supported by multiple lines of computational evidence (ligand-receptor prediction, pathway enrichment, centrality analysis, and spatial correlation), remains entirely predictive at this stage and requires experimental validation through functional assays. While pseudotime analysis infers transcriptional ordering rather than true lineage relationships, our trajectory results suggest that POSTN^+^ CAFs may constitute a plastic, foundational reservoir from which heterogeneous CAF phenotypes potentially emerge, with STAT1 activity potentially serving as a master regulator of this process. The identification of POSTN^+^ CAFs as a subset progressively expanded in advanced-stage disease resonates with growing evidence highlighting matrix-producing fibroblasts as mediators of therapeutic resistance in solid tumors (21, 24, 25). Our trajectory inference extends this paradigm by revealing their progenitor-like positioning at the origin of CAF diversification. This observation is consistent with the interpretation that POSTN^+^ CAFs may constitute a plastic, foundational reservoir from which heterogeneous CAF phenotypes could potentially emerge through transcriptional reprogramming, with STAT1 activity potentially serving as a master regulator of this process. Their spatial confinement to the tumor-stroma interface further underscores a specialized role in delineating the physical and biochemical tumor border, directly contributing to the establishment of an immune-excluded spatial architecture (26).

Within this remodeled stromal compartment, macrophages emerge as pivotal immune collaborators that perpetuate fibroblast-driven immunosuppression (27, 28). The selective association between POSTN^+^ CAFs and APOE^+^ macrophages, rather than other myeloid subsets, indicates a highly specific stromal-immune synergy. APOE^+^ macrophages displayed a distinct M2-like, immunoregulatory state with lipid-associated features, concordant with protumor functions (29, 30). The delineation of MDK-NCL as a central ligand-receptor dyad furnishes a mechanistic basis for this interaction (31). Midkine (MDK) is a multifunctional growth factor involved in cell migration and angiogenesis (32, 33), while nucleolin (NCL) can operate as a cell-surface receptor (34, 35). Their engagement between POSTN^+^ CAFs and APOE^+^ macrophages is likely to reinforce a feed-forward circuit that consolidates both the fibrotic and immunosuppressive attributes of the niche.

This finding requires validation in prostate cancer-specific immunotherapy cohorts—an important avenue for future investigation given the limited immunotherapy response data currently available in prostate cancer, The clinical and translational implications of this axis are of interest and warrant further investigation. Co-infiltration of POSTN^+^ CAFs and APOE^+^ macrophages was consistently associated with poorer prognosis across multiple independent cohorts, underscoring its prognostic robustness. Notably, in the IMvigor210 urothelial carcinoma cohort, this stromal-immune signature was associated with diminished survival benefit from anti-PD-L1 immunotherapy. While these cross-cancer findings are exploratory and require validation in prostate cancer immunotherapy cohorts, they suggest the hypothesis that this axis may have predictive utility for immune checkpoint blockade resistance across solid tumors (36). Such resistance plausibly arises from a dual mechanism: physical T-cell exclusion imposed by the CAF-constructed extracellular-matrix barrier (37), compounded by active immunosuppression mediated by the polarized macrophage compartment (38–40).

While this pan-cancer analysis is descriptive and does not add mechanistic depth beyond the prostate cancer findings, it serves to generate the hypothesis that the POSTN^+^ CAF–APOE^+^ macrophage axis may represent a recurrent stromal-immune feature across solid tumors. In exploratory analyses, our pan-cancer data suggested that the enrichment and spatial co-localization of POSTN^+^ CAFs and APOE^+^ macrophages at the invasive tumor front may represent a recurrent feature across diverse malignancies, including breast, liver, and renal cell carcinomas. While CAF and macrophage states are known to be tissue-specific, the recurrent observation of this axis across tumor types generates the hypothesis that this stromal-immune unit may play a broad role in shaping tumor microenvironments. If validated, therapeutic strategies aimed at disrupting this axis could potentially possess applicability beyond prostate cancer, though tissue-specific optimization would likely be required.

Several caveats regarding our spatial transcriptomic analyses warrant explicit acknowledgment. First, spatial transcriptomic technologies such as Visium and ST provide spot-level resolution (encompassing multiple cells per spot) rather than true single-cell resolution. Consequently, cell-type localization inferred through CellTrek deconvolution represents a computational inference rather than direct observation of individual cell positions. Second, the ligand-receptor interactions inferred from spatial data (e.g., MDK-NCL co-localization) are based on gene expression correlation within spatial neighborhoods and do not demonstrate direct physical binding or functional signaling at the protein level. Third, our spatial analyses were performed on a limited number of prostate cancer sections, and broader tissue-level validation—such as multiplex immunofluorescence staining for POSTN/COL1A1, CD68/APOE, and CD3/CD8—will be essential to confirm the spatial relationships proposed here. These limitations underscore that our spatial findings should be interpreted as supportive, hypothesis-generating evidence rather than definitive proof of *in situ* cellular interactions.

Nevertheless, our study carries certain limitations. Although computational inferences derived from trajectory analysis, cell-cell communication modeling, and spatial correlation offer compelling supportive evidence (41, 42), they necessitate validation through direct functional experimentation. The exact molecular mechanisms through which STAT1 governs the POSTN^+^ CAF state, and how MDK-NCL signaling functionally programs APOE^+^ macrophages, remain to be elucidated using genetic and pharmacological perturbations in experimental models.

Moving forward, this work generates hypotheses for potential stroma-directed combination therapies. The POSTN^+^ CAF–APOE^+^ macrophage axis presents hypothetical actionable nodes for therapeutic exploration, such as inhibiting POSTN-mediated matrix deposition, disrupting MDK-NCL communication, or reprogramming M2-like macrophage polarization. Should these mechanisms be validated experimentally, combining such stromal-modulating agents with immune checkpoint blockade could represent a rational strategy to deconstruct the immune-excluded niche and resensitize treatment-resistant tumors to immunotherapy (43). However, we emphasize that no therapeutic experiments were performed in this study, and these suggestions remain speculative. Future endeavors should prioritize functional validation of this axis in experimental models (e.g., CAF-specific POSTN knockout, MDK-NCL blockade, or macrophage polarization modulation in syngeneic or patient-derived xenograft models), followed by prospective clinical cohorts and the development of targeted inhibitors, before translating this biological insight into therapeutic strategies for patients with advanced prostate cancer and other solid malignancies.

## Data Availability

The original contributions presented in the study are included in the article/**Supplementary Material**. Further inquiries can be directed to the corresponding authors.
