# Supplementary material for: Integrative multi-omics reveals the POSTN+ CAF–APOE+ macrophage axis drives immunosuppression and progression in prostate cancer
Source: Front Immunol. 2026 Aug 10;17:1845274. doi: 10.3389/fimmu.2026.1845274 (PMC13500630; doi:10.3389/fimmu.2026.1845274)
Supplement: Supplementary file 1 [file DataSheet1.docx]

**Full unedited blot for Ref Figure 2**

50kDa

POSTN

β-Tubulin

70kDa

100kDa

POSTN

100kDa

70kDa

β-Tubulin

50kDa
