# Supplementary material for: Integrative multi-omics reveals the POSTN+ CAF–APOE+ macrophage axis drives immunosuppression and progression in prostate cancer
Source: Front Immunol. 2026 Aug 10;17:1845274. doi: 10.3389/fimmu.2026.1845274 (PMC13500630; doi:10.3389/fimmu.2026.1845274)
Supplement: Supplementary file 2 [file Table1.docx]

Supplementary Material

# 1 Supplementary Figures and Tables

## 1.1 Supplementary Table 1. Six CAF subtypes signatures and M1/M2 signatures.

| **iCAF** | LMNA, GSN, DPT, EFEMP1, IL6, C3, COL14A1, CXCL12, TNXB, OGN, PDPN, HAS1, PDGFRA |
| --- | --- |
| **myCAF** | TAGLN, ACTA2, BGN, TPM2, TPM1, MYL9, CTHRC1, POSTN, INHBA, THBS2, MMP11, FAP |
| **apCAF** | CD74, HLA-DRB1, CXCL12, CCL21, HLA-DRA |
| **pCAF** | AURKA, BUB1, CCNB1, CCND1, CCNE1, DEK, E2F1, FEN1, FOXM1, H2AFZ, HMGB2, MCM2, MCM3, MCM4, MCM5, MCM6, MKI67, MYBL2, PCNA, PLK1, TOP2A, TYMS, ZWINT |
| **vCAF** | MYH11, DSTN, MUSTN1, ADIRF |
| **lpCAF** | APOC3, APOC1, APOA2, APOC2, FABP1 |
| **M1 Macrophage Polarization** | IL12, IL23, TNF, IL6, CD86, MHCII, IL1B, MARCO, iNOS, CD64, CD80, CXCR10, CXCL9, CXCL10, CXCL11, IL1A, TNFa, CCL5, IRF5, IRF1, CD40, IDO1, KYNU, CCR7, CD45, CD68, CD115, HLA-DR, CD205, CD14 |
| **M2 Macrophage Polarization** | ARG1, ARG2, IL10, CD32, CD163, CD23, CD200R1, PD-L2, PDL1, MARCO, CSF1R, CD206, IL1RN, IL1R2, IL4R, CCL4, CCL13, CCL20, CCL17, CCL18, CCL22, CCL24, LYVE1, VEGFA, VEGFB, VEGFC, VEGFD, EGF, CTSA, CTSB, CSTC, CTSD, TGFB1, TGFB2, TGFB3, MMP14, MMP19, MMP9, CLEC7A, WNT7B, FASL, TNFSF12, TNFSF8, CD276, VTCN1, MSR1, FN1, IRF4, CD45, CD68, CD115, HLA-DR, CD205, CD14 |

**Supplementary Table 2. Patient information in the single-cell pan-cancer cohort.**

| **Dataset** | Cancer type | No of  samples | Tumor  samples | Normal  samples | No of  cells |
| --- | --- | --- | --- | --- | --- |
| **GSE131907** | LUAD | 22 | 11 | 11 | 84186 |
| **GSE138709** | ICC | 8 | 5 | 3 | 29628 |
| **GSE149614** | LIHC | 18 | 10 | 8 | 57544 |
| **GSE155698** | PDAC | 18 | 15 | 3 | 34057 |
| **GSE159115** | ccRcc | 13 | 7 | 6 | 22208 |
| **GSE163558** | GC | 4 | 3 | 1 | 11638 |
| **GSE166555** | CRC | 25 | 13 | 12 | 67352 |
| **GSE180286** | BRCA | 5 | 5 | 0 | 22548 |
| **GSE181294** | PRAD | 16 | 8 | 8 | 56098 |
| **GSE181919** | HNSC | 29 | 20 | 9 | 36759 |
| **GSE184880** | OVCA | 12 | 7 | 5 | 37367 |

## 1.2 Supplementary Figures

**Supplementary Figure 1.** Overview of the integrated single-cell transcriptomic atlas. **(A)** UMAP colored by cell type and split by tissue origin. **(B)** Pie plot showing cell count distribution across 10 integrated datasets. **(C)** Pie plot showing sample distribution across 10 integrated datasets. **(D)** Stacked bar plot showing cell type proportions in each tissue origin. **(E)** Stacked bar plot showing cell type proportions in each dataset.

**Supplementary Figure 2.** CAF re-clustering analysis in PRAD. **(A)** UMAP showing CAF subpopulations, split by tissue origin. **(B)** UMAP visualizing CAF clustering, with points colored by assigned subtypes. **(C)** Radar plot displaying the distribution of cell cycle states across CAF subtypes. **(D)** Boxplot comparing absolute infiltration levels of CAF subtypes between tumor and normal samples in TCGA-PRAD. **(E)** Radar plot summarizing significantly enriched GO terms from differentially expressed genes across CAF subtypes. **(F)** Heatmap of average expression levels for LOX‑ and MMP‑related genes in each CAF subtype.

**Supplementary Figure3.** Pseudotime analysis of CAF subtypes in PRAD. **(A)** Developmental trajectory of CAFs inferred by Monocle2, overlaid with cell coloring representing distinct CAF subtypes. **(B)** Developmental trajectory of CAFs inferred by Monocle2, colored by sample type. **(C)** Density heatmaps showing the distribution of epithelial-mesenchymal transition (EMT) score (left), hypoxia score (middle), and TGF‑β signaling score (right) across CAF subtypes. **(D)** Dot plots showing Spearman correlations between pseudotime and EMT score (left) or hypoxia score (right), with points colored by CAF subtype. **(E)** Volcano plot of differentially expressed genes between POSTN+ CAFs and other CAF subtypes. Differential expression was assessed using the Wilcoxon rank-sum test.

**Supplementary Figure 4.** Myeloid cells reclustering analysis in PRAD. **(A)** UMAP visualization of key myeloid lineage markers: macrophages (CD163, MRC1, CCR5), monocytes (CD14, FCN1), cDC1s (IDO1, CLEC9A), cDC2s (CD1C, CLEC10A), pDCs (LILRA4, JCHAIN), mDCs (LAMP3), neutrophils (CSF3R, S100A8, S100A9), and proliferation markers (TOP2A, MKI67, STMN1). **(B)** Heatmap displaying the top 5 differentially expressed genes across distinct myeloid subtypes. **(C)** Density plot of IDO1 expression (an M1-associated marker) on UMAP projection. **(D)** Density plot of SPP1 expression (an M2-associated marker) on UMAP projection.

**Supplementary Figure 5.** Analysis of epithelial cell heterogeneity and plasticity in prostate cancer. **(A)** UMAP visualization of epithelial cells, colored by transcriptionally defined epithelial subclusters, depicting cellular heterogeneity. **(B)** UMAP visualization of epithelial cells, colored by tissue type. **(C)** Dot plot showing the expression level and percentage of cells expressing canonical marker genes across epithelial subclusters. **(D)** Heatmap of copy number variation (CNV) across epithelial cells, inferred by inferCNV. **(E)** UMAP visualization of epithelial cells, colored by CNV scores derived from inferCNV analysis for each epithelial subtype. **(F)** Boxplot comparing CNV scores across different tissue types. **(G)** Boxplot comparing CNV scores across epithelial subclusters. **(H)** Scatter plot showing the infiltration proportion of epithelial subtypes across tissue types (Primary, CRPC, mCRPC). P-values were calculated using t-tests for inter-group comparisons. **(I)** Bar plots showing the proportion of each epithelial cell type within each tissue type (top), and the tissue-type composition within each epithelial cell type (bottom). **(J)** Heatmap of Jaccard similarity index for robust epithelial NMF programs derived using the GeneNMF decomposition algorithm. The matrix was systematically clustered, revealing meta-program families (outlined in black). Representative enriched Gene Ontology Biological Process (GOBP) terms for each meta-program family are annotated on the right. **(K)** UMAP feature plots depicting the gene set scores for Meta-Programs 1 through 9 (MP1-MP9). **(L)** Violin plots showing the distribution of gene set scores for Meta-Programs 1 through 9 (MP1-MP9) across epithelial subtypes, calculated using the AddModuleScore function.

**Supplementary Figure 6.** Endothelial cell heterogeneity and functional profiling in PRAD. **(A)** UMAP visualization of transcriptionally defined endothelial cell subclusters, color-coded by subtype identity. **(B)** Dot plot depicting the expression level and cellular prevalence of canonical marker genes across endothelial subtypes. **(C)** Feature plots displaying the spatial expression patterns of subtype-specific marker genes. Expression intensity is scaled, with highest-expressing cells in blue. **(D)** Pie chart representing the tissue-of-origin composition of each endothelial subcluster. **(E)** Heatmap of z-score scaled expression for gene signatures associated with tumor vascularization across endothelial subsets. **(F)** Volcano plot of differentially expressed genes (DEGs) between endothelial cells from CRPC versus primary tumor samples. **(G)** Bar chart illustrating the enrichment of biological pathways derived from upregulated and downregulated DEGs in CRPC endothelial cells.

**Supplementary Figure 7.** Single-cell transcriptional profiling of T and NK cells in PRAD. **(A)** UMAP visualization of T/NK cell subclustering, color-coded by transcriptional subtypes. **(B)** Tissue origin composition of T/NK cell subclusters. **(C)** Expression patterns of selected subtype-specific genes in single-cell resolution, with highest expression shown in blue. **(D)** Violin plots showing functional signature scores (cytotoxicity, terminal-exhaustion, proliferation) across T/NK subclusters. Statistical significance assessed by Kruskal–Wallis test (****p < 0.0001). **(E)** Functional profiling radar chart comparing gene set scores (from TCellSI) across different tissue origins. **(F)** Volcano plot showing differentially expressed genes in T/NK cells between CRPC and primary tumor samples. **(G)** Pathway enrichment analysis of upregulated genes in CRPC T/NK cells. **(H)** Comparison of functional scores in proliferative T cells between primary and CRPC samples by two-sided t-test (p < 0.0001). **(I)** Heatmap showing functional gene expression patterns across T/NK cell subtypes (z-score normalized).

**Supplementary Figure 8.** Single-cell transcriptomic profiling of B cell heterogeneity in PRAD. **(A)** UMAP visualization of transcriptionally defined B cell subclusters, color-coded by subtype identity. **(B)** Tissue-of-origin composition analysis of B cell subtypes, presented as stacked bar plots. **(C)** Feature plots showing the expression of selected specific genes. Cells with the highest expression levels are colored red. **(D)** Dot plot illustrating the cellular prevalence (dot size) and mean expression level (color intensity) of key lineage markers across B cell subclusters. **(E)** Functional enrichment profiling of B cell subclusters via radar plot, highlighting significantly upregulated biological pathways within each cluster. **(F)** Circular composition plot (pie chart) depicting the proportional distribution of B cell subtypes across distinct tissue types.

**Supplementary Figure 9.** High co-infiltration of POSTN+ CAFs and APOE+ macrophages correlates with immunotherapy resistance. **(A)** Spatial characterization in four additional tissue sections. From left to right for each section: SpatialFeaturePlot of the specific gene module signature score, POSTN+ CAF signature score, and APOE+ macrophage signature score. The dot plot on the right shows the Spearman correlation between the spatial signature scores of POSTN+ CAFs and APOE+ macrophages across all spots. **(B)** Enhanced spatial expression profiling via BayesSpace. SpatialFeaturePlots display the expression patterns of POSTN, COL1A1, CD68, APOE, CD3D, CD8B, CD4, CD19, GZMA, and GNLY in tumor tissues from four PRAD patients.

**Supplementary Figure 10.** Pan-Cancer Single‑Cell and Bulk Transcriptomic Landscape of POSTN⁺ CAFs and APOE⁺ Macrophages. **(A)** UMAP projection of CAF subpopulations, colored by POSTN expression group (POSTN⁺ vs POSTN⁻) and faceted by cancer type. **(B)** UMAP visualization of CAF subpopulations, colored by tissue type (tumor vs normal). **(C)** UMAP projection of CAF subpopulations, faceted by tissue origin (tumor/adjacent normal). **(D)** Bar plot showing the proportion of POSTN⁺ CAFs among all CAFs across multiple cancer types, stratified by tissue context. **(E)** UMAP projection of myeloid subpopulations, colored by APOE expression group (APOE⁺ vs APOE⁻) and faceted by cancer type. **(F)** UMAP visualization of myeloid subpopulations, colored by tissue type (tumor vs normal). **(G)** UMAP projection of myeloid subpopulations, faceted by tissue origin (tumor/adjacent normal). **(H)** Bar plot showing the proportion of APOE⁺ macrophages among all myeloid cells across multiple cancer types, stratified by tissue context. **(I)** Kaplan–Meier survival analysis in the TCGA pan‑cancer cohort, stratified by combined infiltration of POSTN⁺ CAFs and APOE⁺ macrophages (low vs high co‑infiltration).

**Supplementary Figure 11.** Spatial distribution of POSTN and APOE at tumor–stroma interfaces across multiple cancer types. **(A-G)** Pan-cancer spatial characterization of POSTN and APOE expression at the tumor-stroma interface. For each cancer type—**(A)** BRCA, **(B)** CESC, **(C)** COAD, **(D)** GBM, **(E)** HNSC, **(F)** LIHC, **(G)** KIRC—the following analyses are shown from left to right per tissue section: Left, SpatialDimPlot displaying SpaCET-derived regional annotations (Tumor [Mal; red], Interface [Bdy; yellow], and Stroma [nMal; green] spots); Middle, SpatialFeaturePlots showing expression of POSTN (top) and APOE (bottom); Right, Bar chart comparing the average expression levels of POSTN and APOE across tumor, interface, and stromal compartments.

**Supplementary Figure 12.** Multiplex immunofluorescence of paired normal and tumor prostate tissues. POSTN (green) shows strong network-like staining in periglandular tumor stroma, overlapping with CD68⁺ macrophages (red), while CD8⁺ T cells (magenta) are largely excluded from these regions. DAPI (blue), nuclear counterstain. Scale bars, 50 μm.
